# A capillary electrophoresis-based multiplex PCR assay for expanded carrier screening in the eastern Han Chinese population

**DOI:** 10.1038/s41525-021-00280-y

**Published:** 2022-01-25

**Authors:** Ping Hu, Jianxin Tan, Feng Yu, Binbin Shao, Fang Zhang, Jingjing Zhang, Yingchun Lin, Tao Tao, Lili Jiang, Zhengwen Jiang, Zhengfeng Xu

**Affiliations:** 1 Department of Prenatal Diagnosis, State Key Laboratory of Reproductive Medicine, Women’s Hospital of Nanjing Medical University, Nanjing Maternity and Child Health Care Hospital, Nanjing, 210004 Jiangsu People’s Republic of China; 2 Genesky Diagnostics (Suzhou) Inc, Suzhou, 215123 Jiangsu People’s Republic of China

**Keywords:** Molecular medicine, Preventive medicine

## Abstract

Expanded carrier screening, a type of reproductive genetic testing for couples, has gained tremendous popularity for assessing the risk of passing on certain genetic conditions to offspring. Here, a carrier screening assay for 448 pathogenic variants was developed using capillary electrophoresis-based multiplex PCR technology. The capillary electrophoresis-based multiplex PCR assay achieved a sensitivity, specificity, and accuracy of 97.4%, 100%, and 99.6%, respectively, in detecting the specific variants. Among the 1915 couples (3830 individuals), 708 individuals (18.5%) were identified as carriers for at least one condition. Of the 708 carriers, 633 (89.4%) were heterozygous for one condition, 71 (10.0%) for two disorders, 3 (0.4%) for three disorders, and 1 (0.1%) for four disorders. Meanwhile, 30 (1.57%) couples were identified as at‐risk couples. This study describes an inexpensive and effective method for expanded carrier screening. The simplicity and accuracy of this approach will facilitate the clinical implementation of expanded carrier screening.

## Introduction

Expanded carrier screening (ECS) represents a type of reproductive genetic testing for couples, which aims to identify asymptomatic carriers for a broad array of specific genetic (autosomal or X-linked) disorders either when pregnancy or planning to become pregnant. This test enables the couples to learn the likelihood of having an affected offspring, regardless of ethnic background, race, or family history^1^. Compared with traditional carrier screening, ECS includes a much larger number of inherited genetic conditions and thus identifies a higher proportion of at‐risk couples in the general population in a cost-effective way^2,3^. Therefore, ECS has gained tremendous popularity and has been recommended by many professional societies^4–6^.

Multiple high-throughput platforms, including microarray^7^ and next generation sequencing (NGS)^8^, have been used for ECS since its introduction into clinical practice in 2011. In recent years, NGS has become a unifying platform for ECS because of its capacity to identify rare or novel pathogenic variants and to analyze multiple genes and multiple samples simultaneously in a cost-effective manner^9^. Several reports have demonstrated the excellent sensitivity, specificity and feasibility of NGS for carrier screening^10,11^. Nonetheless, there are a few limitations of NGS that may constrain the value of ECS. Currently, difficulties in interpretation of numerous sequencing variants represent the biggest stumbling block to a large-scale implementation of NGS-based ECS^12^. Furthermore, some certain genes of high clinical importance are technically challenging to assess with NGS because of pseudogenes, CGG repeat expansions, or DNA structural variations (e.g., survival of motor neuron 1 (*SMN1*) [MIM *600354] for spinal muscular atrophy (SMA) and fragile X mental retardation 1 (*FMR1*) [MIM *309550] for fragile X syndrome)^13^. Although technological advances have led to a sharp decrease in sequencing costs, the current cost of NGS-based ECS, ranging from approximately $200 for tens of genes to $500 for hundreds of genes per couple^14^, precludes its clinical utility particularly in most of the developing world.

Capillary electrophoresis is a high-throughput separating technique commonly employed for DNA sequencing analysis due to high resolution, short run times, and minimal space requirements^15^. The present study established a capillary electrophoresis-based multiplex PCR assay (CEBMPA) for carrier screening to simultaneously analyze 448 disease-causing variants among 24 genes associated with 20 conditions, which covers the most common pathogenic variants of these genes in the Chinese population. This screening system not only produces results that are easy to be interpreted, but it also allows genotyping some genes with pseudogenes, structural variation, or repeat expansion, thus potentially offering an inexpensive and effective approach for ECS.

## Results

### Validation study

**Fig. 1:**
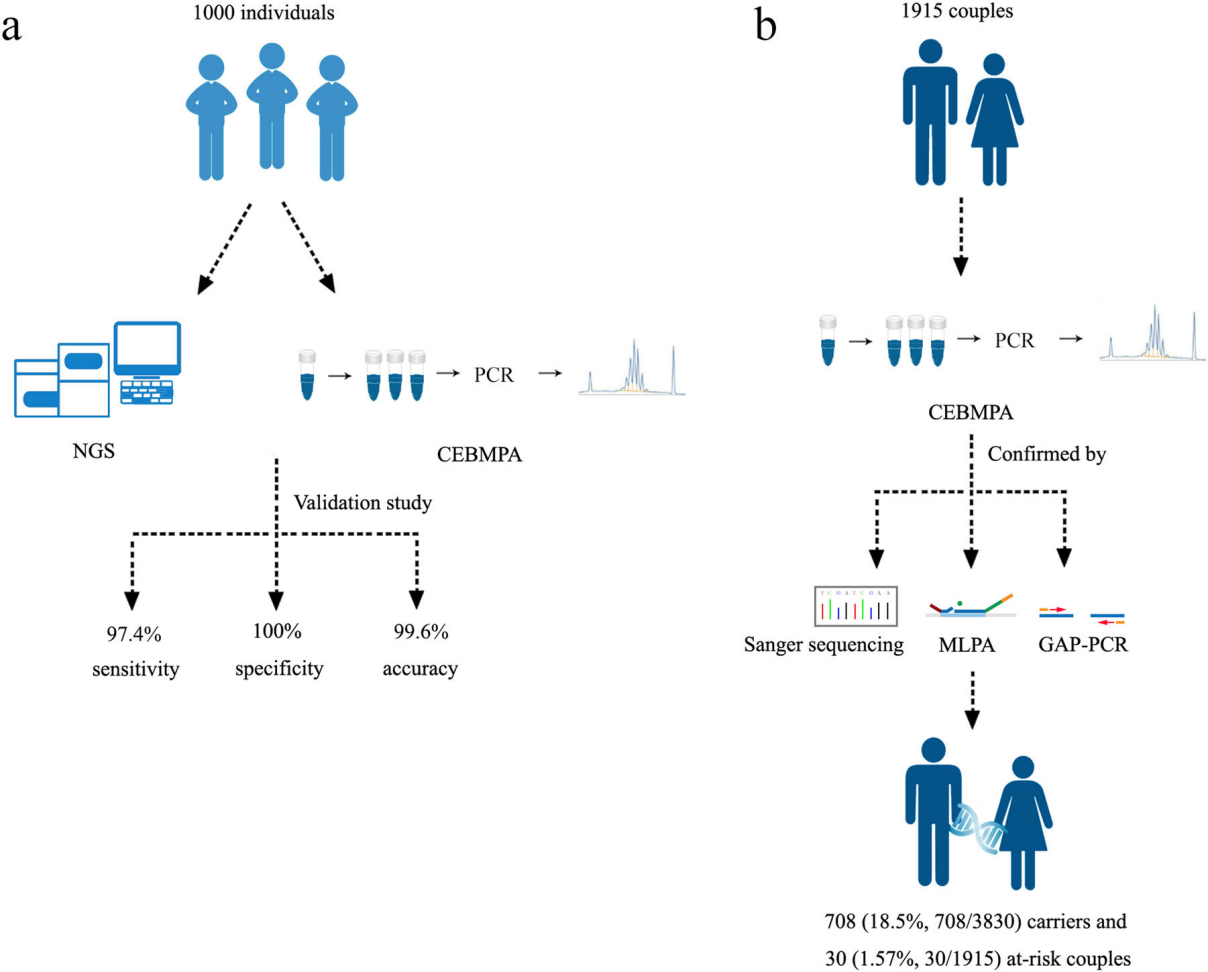
The flow diagram depicts the recruitment of participants and methods used for screening. Two participant cohorts were used in this study. **a** The first participant cohort consists of 1000 individuals, who were detected in parallel with NGS and CEBMPA to validate the sensitivity and specificity of CEBMPA. **b** The second cohort including 1915 couples (3830 individuals) were detected with CEBMPA. All detected variants were confirmed by alternative methods, such as Sanger sequencing, multiplex ligation-dependent probe amplification, or Gap-PCR.

Flow diagrams showing the recruitment of participants and methods used for screening are depicted in Fig. 1. First, 1000 individuals in the initial participant cohort were assessed in parallel with both NGS and CEBMPA. To validate the sensitivity and specificity of CEBMPA, variants in overlapping genes imputed by the two methods were compared (Table 1). In total, CEBMPA and NGS identified 152 and 156 variants, respectively, in the ten overlapping genes. Specifically, four variants that were not included in screening panel of CEBMPA were identified by NGS but omitted by CEBMPA: *MMACHC* (NM_015506.3), c.440_441delGT; *MMUT* (NM_000255.3), c.1975C>T and c.1663G>A; and *GJB2* (NM_004004.5), c.2T>C. Notably, no false positive results were observed from either NGS or CEBMPA. Compared with NGS, CEBMPA achieved a sensitivity, specificity, and accuracy of 97.4%, 100%, and 99.6%, respectively, in detecting the specific variants. The sensitivity (detection rate), specificity, and accuracy of CEBMPA in detecting each gene are presented in Table 1.

**Table 1:** Number of variants in overlapping genes imputed by NGS and CEBMPA in 1000 individuals.

Diseases	Genes	Number of pathogenic variants		CEBMPA		
		CEBMPA	NGS	Sensitivity (Detection rate)	Specificity	Accuracy
Genetic deafness	*GJB2*	34	35	97.1%	100%	99.9%
Hepatolenticular degeneration	*ATP7B*	26	26	100%	100%	100%
Genetic deafness	*SLC26A4*	26	26	100%	100%	100%
Phenylketonuria	*PAH*	20	20	100%	100%	100%
α-Thalassemia	*HBA*	18	18	100%	100%	100%
Methylmalonic acidemia with homocystinuria Cb1C	*MMACHC*	15	16	93.8%	100%	99.9%
Methylmalonic acidemia	*MUT*	5	7	71.4%	100%	99.8%
Tetrahydrobiopterin deficiency	*PTS*	5	5	100%	100%	100%
β-Thalassemia	*HBB*	3	3	100%	100%	100%
Genetic deafness	*MT-RNR1*	0	0			

### Carrier frequencies

Next, the second cohort of 1915 couples (3830 individuals) were assessed using CEBMPA. All detected variants were confirmed by alternative methods, such as Sanger sequencing, multiplex ligation-dependent probe amplification, or Gap-PCR. A total of 100% concordance between CEBMPA and alternative methods was obtained. Among the 3830 individuals (1915 couples), 134 (29.9%, 134/448) different variants located in 21 genes were identified (Supplementary Table 4). Variants in the *RBM8A, SLC3A1*, and *PREPL* genes were not observed in any tested individuals.

**Fig. 2:**
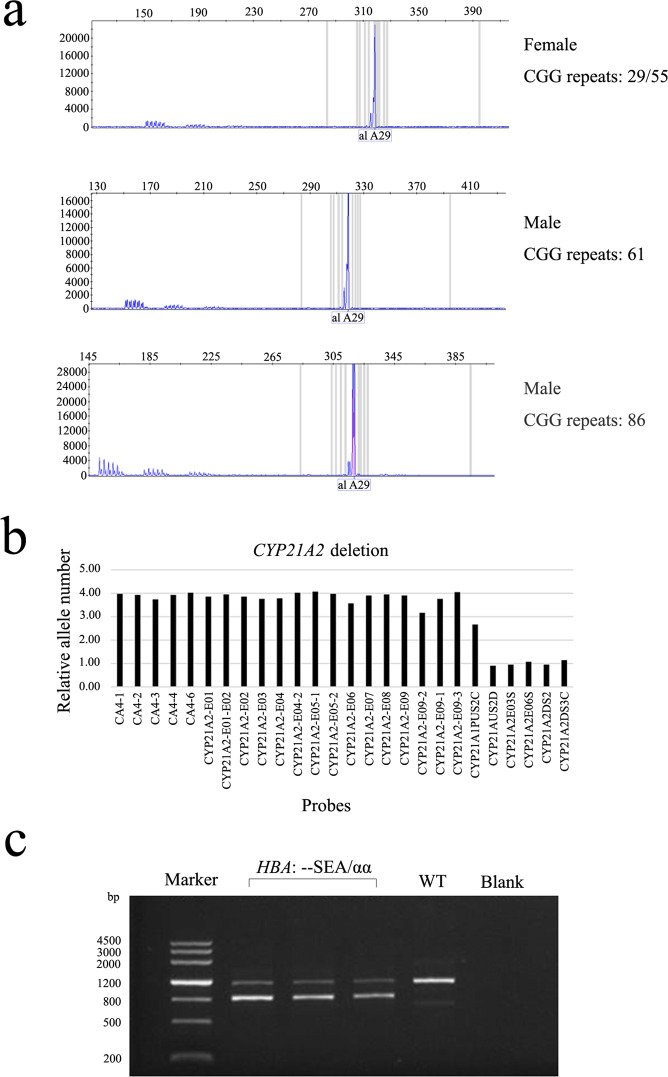
Representative images of variants detected by different methods. **a** Confirmation of *FMR1* CGG repeats in three carriers with premutation using the Asuragen AmplideX™ FMR1 PCR Kit. The image showing hexachloto-Fluorescein (HEX) channel from capillary electrophoresis. **b** Analysis of *CYP21A2* deletion by using HLPA. The shown columns are relative allele number of *CYP21A2* exons. **c** Gap-PCR analysis was performed to analyze of *HBA1* deletions. A representative gel image of banding pattern observed for *HBA1* --(SEA) deletion is shown. WT wide type.

Furthermore, 18.5% of individuals (*n* = 708) were identified as carriers for at least one condition. The carrier frequencies for the tested diseases are shown in Table 2, and representative images for several selected variants detected with different methods are presented in Fig. 2. *GJB2*-related non-syndromic hearing loss with a frequency of 3.7% was the most common autosomal recessive disease, followed by congenital adrenal hyperplasia (CAH) (2.9%) and hepatolenticular degeneration (2.6%). Of the 1915 female participants, 14 were found to be heterozygous for X-linked disorders. The most common X-linked disease was X-linked ichthyosis (*n* = 5), followed by Duchenne muscular dystrophy (*n* = 2) and Int22h1/Int22h2 mediated chromosome Xq28 duplication (*n* = 2).

**Table 2:** Carrier frequencies for autosomal diseases in 1915 couples (3830 individuals).

Inheritance patterns	Autosomal diseases	Genes	Number of pathogenic variants	Carrier frequencies (1/*n*)	Number of at-risk couples
Autosomal recessive	Genetic deafness	*GJB2*	141	27	6
	Congenital adrenal hyperplasia	*CYP21A2*	114	34	1
	Hepatolenticular degeneration	*ATP7B*	100	38	0
	Genetic deafness	*SLC26A4*	91	42	0
	Spinal muscular atrophy	*SMN1*	85	45	4
	α-Thalassemia	*HBA*	84	46	0
	Phenylketonuria	*PAH*	69	56	1
	Methylmalonic acidemia with homocystinuria Cb1C	*MMACHC*	41	93	1
	Tetrahydrobiopterin deficiency	*PTS*	21	182	0
	Methylmalonic acidemia	*MMUT*	11	348	0
	β-Thalassemia	*HBB*	10	383	0
	Thrombocytopenia-absent radius syndrome	*RBM8A*	0	0	0
	Hypotonia-cystinuria syndrome	*SLC3A1, PREPL*	0	0	0
X-linked recessive	X-linked ichthyosis	*STS*	5	383	5
	Duchenne muscular dystrophy	*DMD*	2	958	2
	Int22h1/Int22h2 mediated chromosome Xq28 duplication syndrome	Xq28	2	958	2
	Fragile X syndrome	*FMR1*	1^a^	1915	1
	Hemophilia A	*F8*	1	1915	1
	Xp11.22 microduplication syndrome	*HUWE1*	1	1915	1
	Pelizaeus-Merzbacher disease	*PLP1*	0	0	0
Mitochondrial inheritance	Genetic deafness	*MT-RNR1*	6	638	5
In total			785	4.9	30

^a^Two men with FMR1 pre-mutation were not included.

Notably, CEBMPA was able to detect some genes with pseudogenes or repeat expansion that cannot be detected by NGS. Of the 3830 individuals (1915 couples), 85 (2.2%, 85/3830) were identified to have a heterozygous deletion of the *SMN1* gene, and an *FMR1* premutation was identified in two men and one woman (0.8‰, 3/3830). In addition, 114 variants were identified in the *CYP21A2* gene.

Of the 708 identified carriers, 633 (89.4%) were heterozygous for one condition, 71 (10.0%) for two disorders, 3 (0.4%) for three disorders, and 1 (0.1%) for four disorders. Furthermore, 16 individuals were found to be homozygous or compound heterozygous for the following conditions: hepatolenticular degeneration (*n* = 10), *GJB2*-related non-syndromic hearing loss (*n* = 3), phenylketonuria (*n* = 1), and CAH (*n* = 2).

### Allele frequencies

**Fig. 3:**
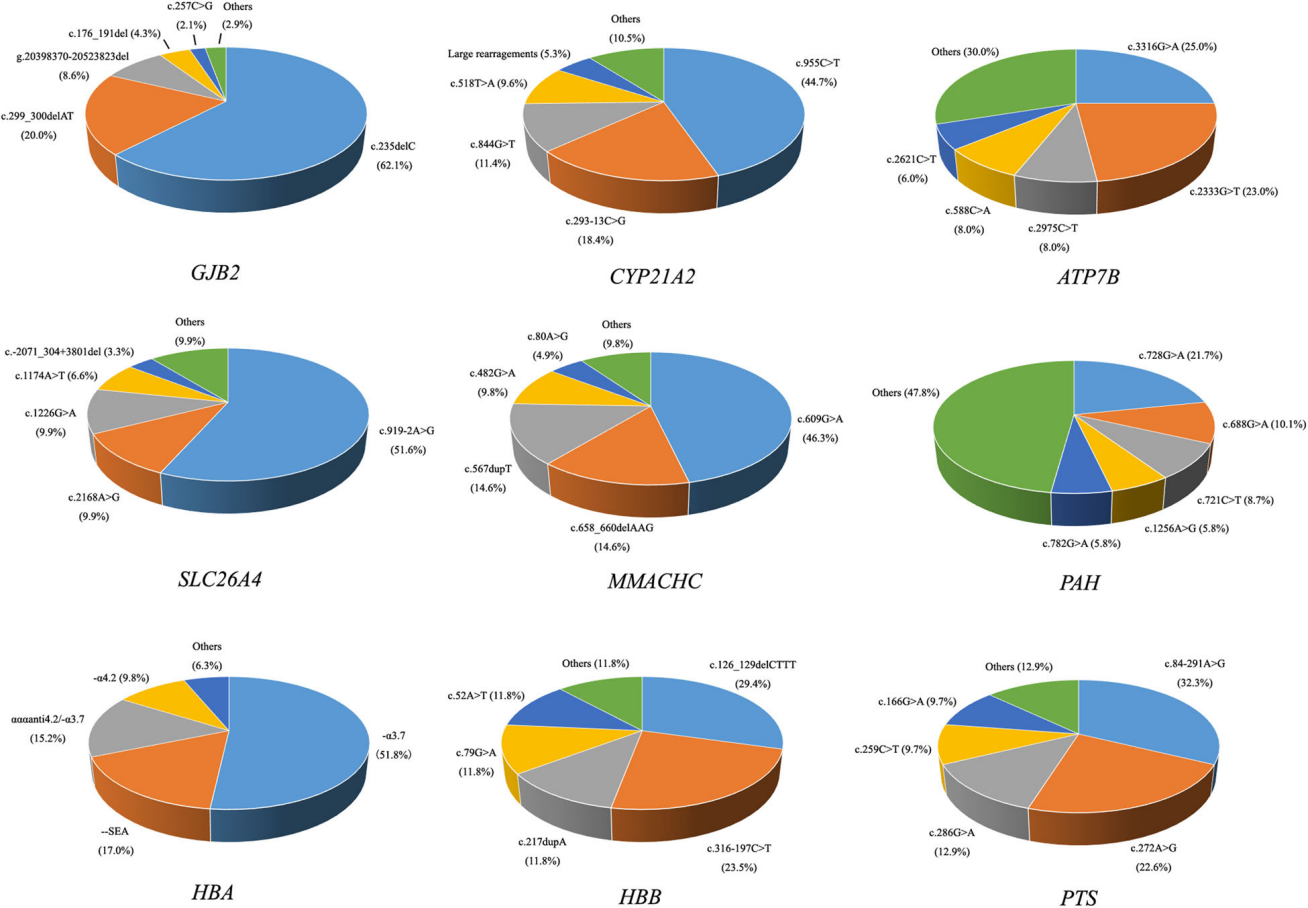
The percentages of each variant in nine genes detected by CEBMPA. The mutational spectrum of *GJB2, CYP21A2, ATP7B, SLC26A4, MMACHC, PAH, HBA, HBB* and *PTS* in 1915 couples (3830 individuals).

The mutational spectrum of *GJB2, CYP21A2, ATP7B, SLC26A4, MMACHC, PAH, HBA, HBB*, and *PTS* is shown in Fig. 3. A total of 140 subjects were identified as *GJB2* carriers with the most common mutation being c.235delC. A total of 91 individuals were identified as carriers for a mutation of *SLC26A4*. c.919-2A>G was the most frequent hot-spot mutation with an allele frequency of 1.2% (47/3830), and c.2168A>G was the second-most frequent hot-spot mutation. A total of 69 subjects were identified as mutated *PAH* carriers. Mutations of *PAH* were distributed across all exons. The most frequent *PAH* variant was c.728G>A (21.7%), followed by c.688G>A (10.1%), c.721C>T (8.7%), c.1256A>G (5.8%), and c.721C>T (5.8%). The most common variant for *PTS* was c.84-291A>G (32.3%), followed by c.272A>G (22.6%), c.286G>A (12.9%), and c.259C>T (9.7%). The mutational spectrum of *PAH, GJB2, SLC26A4*, and *PTS* in this study is consistent with previous reports on the Chinese population^16–19^. In addition, c.609G>A was the most frequent mutation for *MMACHC*, which agrees with a previous report on Chinese patients with methylmalonic acidemia^20^. The most common two deletions of *HBA* were ‐α3.7 (51.8%) and ‐‐SEA (17.0%), and the two most common mutations of *HBB* were c.126_129delCTTT (29.4%) and c.316-197C>T (23.5%). The top-ranked variants for thalassemia in the study population were similar to those found in southern China^21^. A deletion of exon 7 is the most common mutation of *SMN1* in this study population (100%), which agrees with previous findings^22^.

The most frequent variant for *CYP21A2* was c.955C>T (44.7%), followed by c.293-13C>G (18.4%), c.844G>T (11.4%), c.518T>A (9.6%), and large rearrangements (5.3%). Furthermore, c.3316G>A was the most common variant and c.2333G>T was the second most frequent mutation for *ATP7B* in this study cohort. This mutational spectrum of *CYP21A2* and *ATP7B* is not consistent with reports from previous studies on Chinese patients^23,24^. Additionally, six individuals (1.6‰, 6/3830, Table 3) were identified as carriers of *MT-RNR1*.

**Table 3:** Six carriers of *MT-RNR1* mutations.

Mutation	Gender	Number of carriers
m.1555A>G, homoplasmy	Female	3
m.1555A>G, homoplasmy	Male	1
m.1494C>T, homoplasmy	Female	2

### Carrier couples

Of the 1915 couples, 30 were identified as at‐risk couples, among which 13 couples were carriers for pathogenic variants in the same autosomal gene and 17 women were carriers for X-linked or mitochondrial diseases. Table 2 lists the disorders identified in the at-risk couples who may pass these disorders to their offspring. Unsurprisingly, the most common disorder identified in the carrier couples was *GJB2*-related non-syndromic hearing loss (*n* = 6), followed by X-linked ichthyosis (*n* = 5) and mitochondrial deafness (*n* = 5).

## Discussion

The present study successfully established a method, known as CEBMPA, for carrier screening, which simultaneously genotypes 448 disease-causing variants among 24 genes associated with 20 conditions in the Chinese population. The data show that CEBMPA exhibited excellent sensitivity and specificity. Overall, 18.5% of tested individuals (*n* = 708) were identified as carriers for at least one condition, and 30 (1.57%, 30/1915) couples were identified as at‐risk couples, among which 13 (43.3%, 13/30) couples were carriers for the same autosomal gene and 17 (56.7%, 17/30) women were carriers for X-linked or mitochondrial diseases.

Notably, several conditions that have high clinical importance but are technically challenging to assess by NGS were also included in this study, such as SMA, Fragile X syndrome, and CAH. Among them, carrier screening for SMA and Fragile X syndrome has been recommended by the American College of Obstetricians and Gynecologists^25^. In the present study, the carrier frequency for SMA was 2.2%, which is comparable to previously reported in the Chinese population (1.7–2.3%)^26^. Furthermore, the incidence of CAH has been reported to as 1 in 6084 in China^27^, with an estimated carrier frequency of 1 in 39. The carrier frequency for the variants involved in CAH observed in this study was 1 in 34. The prevalence of *FMR1* pre-mutation in this cohort (1 in 1915) is similar to that reported in pregnant women from Hong Kong (1 in 1325)^28^ or Taiwan (1 in 1955)^29^ in southern China. Overall, the present study found carrier frequencies similar to those previously reported in Chinese populations, suggesting that CEBMPA is a reliable approach for detecting genes with pseudogenes or CGG repeat expansion.

NGS is increasingly being embraced and has become a unifying platform for ECS^9^. Compared with targeted genotyping approach, NGS allows to identify many more variants, yields higher detection rates of carrier individuals, and reduces the residual risks^10^. Furthermore, NGS continuously accumulates data on the variant lists based on new findings and reclassification, provides a more homogeneous pan-ethnic investigation of the gene-disease pair, and has a higher sensitivity for consanguineous couples with private variants^30^. NGS may identify many variants of uncertain significance. This difficulty is currently circumvented by a streamlined bioinformatic pipeline that only returns pathogenic/likely pathogenic variants^31^. However, variant interpretation for NGS is still challenging in many clinical laboratories^12^. Variabilities in variant interpretation will confuse clinicians and increase both the posttest genetic counseling workload and patient’s anxiety. The genetic counseling workload in China is already extremely heavy due to the largest population in the world and a shortage of qualified genetic counseling staff^32^. Meanwhile, the assurance of pathogenic variants by clinical laboratories can lead to higher overall costs^14^. To establish a feasible method for ECS in the Chinese population, this study used CEBMPA to genotype a set of predefined pathogenic variants. This approach produces results that are easy to be interpreted and will facilitate posttest genetic counseling.

Currently, it is generally accepted that disease severity is an important factor for carrier screening^33^. However, different pathogenic variants in the same gene may cause a spectrum of mild to severe phenotype. For example, the *PAH* c.158G>A (p. Arg53His) variant leads to mild hyperphenylalaninemia, which has a slight increase in Phe levels and requires no treatment^34,35^. Moreover, homozygous of this variant or in cis with other pathogenic variants was observed in healthy individuals^35^. The pathogenicity of *GJB2* c.109G>A (p. Val37Ile) variant is conflicting interpretation, and this variant was observed to be homozygous in healthy subjects and had an allele frequency of 10% in Eastern Asia population^36^. These two variants, along with other variants known to cause mild phenotype or associated with conflicting interpretations, were intentionally excluded from the carrier screening panel in the present study.

A recent survey by Nijmeijer et al. in the Netherlands showed that cost is one of the most important reasons for participants to decline ECS^37^. In the US, over half of respondents would undergo ECS only if the test was covered by insurance, and a majority were willing to pay up to only $50–$100^38^. Therefore, cost will likely be a deciding factor for the successful implementation of ECS in the general population^39^. In the present study, the cost for CEBMPA was dramatically lower than that for NGS ($170 vs. $340 per couple). In addition, we are currently designing and optimizing an expanded CEBMPA test to screen approximately 200 genetic conditions. After optimization, the cost of the expanded CEBMPA test is expected to be similar with that of the CEBMPA test with 20 conditions (~$200 vs. $170 per couple). CEBMPA offers an alternative and inexpensive technology for ECS; as such, it may be particularly useful in economically underdeveloped regions. The key strength of this study is the successful establishment of an inexpensive and effective method for ECS. As validated by NGS, this method showed excellent sensitivity and specificity in genotyping many predefined disease-causing variants. Therefore, it is a viable alternative for clinical implementation of ECS owing to its advantages, as it is easy to be interpreted and allows genotyping some genes with a triplet repeat region or large rearrangements. In addition, this study reports the carrier frequencies of several disorders in the Chinese population.

Currently, a limited panel with tens of conditions is preferred than an expanded panel with hundreds of conditions for carrier screening in China due to the following reasons. First, screening a larger number of conditions will identify more individuals as carriers, who should be offered adequate posttest genetic counseling to ensure that they understand the actual results in terms of their genetic implications^40^. In China, there is a severe shortage of genetic counselors to meet the posttest requirements for an expanded panel^41^. Second, cost is one of the most important factors for a successful carrier screening program. It is suggested that the cost of the screening program should be as low as possible in China^41^. The cost of an ECS panel is often higher than that of a limited panel. The high cost may result in a low level of acceptance of the screening program in China^42^. Third, the prevalence of many rare diseases and the mutation spectra of disease genes in the Chinese population remain uncertain^41^. Certain rare genetic conditions screened may make difficulties in assessing residual risk and increase genetic counseling burden and patient’s anxiety^43^. Altogether, since carrier screening is still in its infant stage in China, panels with ~10–20 genetic conditions are considered desirable at this stage^44,45^.

This study has some limitations worth noting. First, the sample size in this study is relatively small, particularly in the validation study. The small sample size (1000 individuals) may be insufficient to identify many additional variants that can be detected by NGS and missed by CEBMPA, because the allele frequency of many pathogenic variants commonly encountered is far below 1/1000. To enhance the statistical power, we will compare genotypes from CEBMPA to those from whole-exome sequencing datasets in the Han Chinese population in the future studies. Most of the subjects in the study population were from the Han ethnicity. According to the most recent National Population Census in 2010, Han ethnicity accounts for 91.6% of the overall Chinese population, and the other 56 ethnic minority groups (8.4%) primarily reside in the northern, southern, and western frontier of China^46^. Thus, CEBMPA should be applicable to most of the Chinese population, particularly those in eastern China. Second, the variants of RBM8A, SLC3A1 and PREPL for hypotonia-cystinuria syndrome were not observed in this study population, and five mutations identified by NGS were not included in the CEBMPA screening panel. These results suggest that the variants and genes included in the carrier screening panel need to be optimized. Future studies will collect subjects from different ethnic groups and improve the carrier screening panel. Third, CEBMPA is a closed system that should be re-designed and validated clinical targeted panels when new pathogenic variants or genes need to be incorporated into. Fourth, CEBMPA is a targeted genotyping approach that fails to identify private and novel pathogenic variants in several relevant genes for ECS. This may lead to carrier missing and increased residual risks, which need to be emphasized in both pretest and posttest counselling. In our screening system, women planning pregnancy or below 20 gestational weeks received pretest education via leaflets and an online video^39^, and those who were interested in the test were counselled to explain methods, turnaround time, potential risks, benefits, and limitations of the testing with emphasizing the residual risks.

This study provides an inexpensive and effective method for ECS, which produces results that are easy to be interpreted and allows for genotyping some genes with a triplet repeat region or large rearrangements. The simplicity and accuracy of this approach will facilitate the clinical implementation of ECS.

## Methods

### Participants

A total of 4830 blood samples were collected from participants who underwent carrier screening for SMA at Department of Prenatal Diagnosis, Nanjing Maternity and Child Health Care Hospital. The study population included 1000 individuals and 1915 couples with an average age of 30.1 years and a median age of 29 years. Most participants were from Jiangsu or Anhui Province in eastern China. The present study used two participant cohorts for the following purposes: one was assessed with both NGS and CEBMPA to validate the sensitivity and specificity of CEBMPA, and the other was assessed with CEBMPA to calculate the frequencies of at-risk couples per condition. The first participant cohort consisted of 1000 individuals. The majority of these individuals in this cohort were pregnant women. The second cohort exclusively included 1915 couples (3830 individuals). Informed consent was obtained from all study participants at the time of providing a blood sample. Genomic DNA isolation was performed using an Automated Nucleic Acid Extractor (RBC Bioscience, New Taipei City, Taiwan). This study was compliant with the Guidance of the Ministry of Science and Technology for the Review and Approval of Human Genetic Resources. All study procedures were approved by the Ethical Committee of Nanjing Maternity and Child Health Care Hospital in accordance with the Helsinki Declaration of 1975, as revised in 2000.

### Capillary electrophoresis-based multiplex platform

CEBMPA, composed of SNaPshot, Multiplex Fluorescent PCR, AccuCopy quantification, and high-throughput ligation-dependent probe amplification (HLPA), was used to detect 448 disease-causing variants among 24 genes associated with 20 conditions. The selection of these conditions was based on the long-standing criteria for carrier screening initially described by Wilson and Jungner^47^. All the assessed variants were selected based on a literature review and local database (the incidences of genetic diseases and the severity of mutation phenotypes) in combination with additional databases such as ClinVar and Human Gene Mutation Database. The genes, variants, and detection methods used for the 24 tested genes are listed in Table 4 and Supplementary Table 1.

**Table 4:** The gene list and number of variants detected by CEBMPA.

Diseases	Genes	Number of variants
Genetic deafness	*GJB2* (NM_004004.5)	23
	*SLC26A4* (NM_000441.1)	60
	*MT-RNR1* (NC_012920)	2
α-Thalassemia	*HBA1/HBA2*	5
	*HBA1* (NM_000558.3)	3
	*HBA2* (NM_000517.4)	9
β-Thalassemia	*HBB* (NM_000518.4)	58
Duchenne muscular dystrophy	*DMD* (NM_004006.2)	1
Hemophilia A	*F8* (NM_000132.3)	23
Fragile X syndrome	*FMR1* (NM_002024.5)	1
X-linked ichthyosis	*STS* (NM_000351.4)	1
Spinal muscular atrophy	*SMN1* (NM_000344.3)	7
Phenylketonuria	*PAH* (NM_000277.1)	96
Tetrahydrobiopterin deficiency	*PTS* (NM_000317.2)	13
Methylmalonic acidemia	*MMUT* (NM_000255.3)	25
Methylmalonic acidemia with homocystinuria Cb1C	*MMACHC* (NM_015506.2)	19
Congenital adrenal hyperplasia	*CYP21A2* (NM_000500.7)	23
Hepatolenticular degeneration	*ATP7B* (NM_000053.3)	70
Thrombocytopenia-absent radius syndrome	*RBM8A* (NM_005105)	3
Hypotonia-cystinuria syndrome	*SLC3A1* (NM_000341.3);*PREPL* (NM_006036.4)	1
Xp11.22 microduplication syndrome	*HUWE1* (NM_031407.6)	1
Pelizaeus-Merzbacher disease	*PLP1* (NM_000533.4)	2
MECP2 duplication syndrome	*MECP2* (NM_004992.3)	1
Int22h1/Int22h2 mediated chromosome Xq28 duplication syndrome	Xq28 (154.896 Mb–155.335 Mb)	1

NM accession number is a unique gene identifier that links to the GenBank record.

SNaPshot reactions, as assessed by an ABI SNaPshot™ Multiplex Kit (Applied Biosystems, Foster, CA, USA) and specific primers, were used for screening the single nucleotide mutations of *ATP7B, MMACHC, MUT, PAH, PTS, HBA1, HBB, CYP21A2*, and *F8*. For the *F8* gene, AccuCopy quantification combined with two multiplex pre-amplifications of long-distance PCR method was performed to detect intron 22 inversion^48^. Briefly, PCR reactions were performed in a total volume of 20 μl containing 1 × GC Buffer I, 0.2 mM dNTPs, 7.5% dimethyl sulfoxide, 0.2 µM of each primer, and 0.6 unit of LA Taq Hot Start DNA polymerase (Takara, Dalian, China). Each reaction initiated with a denaturation step at 98 °C for 1 min, followed by 30 cycles of 10 s at 98 °C, 15 min at 68 °C, and completed with 72 °C for 10 min. The PCR products underwent a multiplex PCR to amplify three markers, four references (2p, 10p, 16p, and 20q) and a sex-typing fragment. The final PCR products were loaded onto ABI 3730xL DNA Analyzer (Applied Biosystems).

*GJB2, MT-RNR1*, and *SLC26A4* mutations were genotyped by using single nucleotide polymorphism genotyping with an improved multiplex ligation detection reaction technique^49^. In brief, PCR reaction mixture (20 μl) was prepared as follows: 1 × GC Buffer I (Takara), 0.3 mM of each dNTP, 3.0 mM MgCl_2_, 1 unit Hot-Start Taq DNA polymerase (Takara), 0.2 µM of each primer, and 20 ng of genomic DNA. Thermal cycle conditions were as follows: an initial incubation at 95 °C for 2 min; 11 cycles of 20 s at 94 °C, ramped down from 65 °C for 40 s at a speed of 0.5 °C/cycle, and 90 s at 72 °C; 24 cycles of 20 s at 94 °C, 30 s at 59 °C, and 90 s at 72 °C; and 2 min at 72 °C. Equivalent amounts of PCR products were mixed, purified by digestion with 1 unit of shrimp alkaline phosphatase for 1 h at 37 °C, and incubated at 75 °C for 15 min to deactivate the phosphatase. Subsequently, ligation reaction mixture (20 μl) was prepared as follows: 1 × ligation buffer, 80 units Taq DNA ligase (New England Biolabs, Beverly, MA, USA), 2 μl of probe mixture, 1 μl of labeling oligo mixture, and 5 μl of purified PCR products. The ligation protocol was an initial denaturation at 98 °C for 3 min, followed by 38 cycles of 1 min at 94 °C, 4 min at 56 °C. The final products were subjected to sequence analysis on ABI 3730xL DNA Analyzer (Applied Biosystems).

Large deletions or duplications in *DMD, CYP21A2, F8, STS, SMN1* (including six single nucleotide mutations), *HBA1/2, HBB, RBM8A, SLC3A1/PREPL, HUWE1, PLP1, MECP2*, and Xq28 were analyzed by HLPA^50,51^. HLPA was modified from multiplex ligation-dependent probe amplification (MLPA) by introducing a lengthening ligation system and four types of fluorophore-labeled 5′ universal primers together with two types of 3′ primers to quantify up to 200 genomic loci in a single PCR test^50^. Roughly, 200 ng of genomic DNA (in 10 μl Tris-EDTA buffer, pH 8.0) was heated at 98 °C for 6 min and then added with 10 μl of ligation mixture containing 0.5 μl of Taq ligase (Takara), 2 μl of 10 × Taq Ligase buffer, 1 μl of 20 × Probe Mixture and 7.5 μl of double distilled H_2_O. The ligation was carried out using the following thermal cycle conditions: 4 cycles of 1 min at 94 °C, 4 h at 60 °C; 2 min at 94 °C, and hold at 72 °C. Thereafter, 20 μl of 2 × Stop Buffer was added to terminate the reaction. PCR amplification was prepared in a total volume of 20 μl containing 1 × Taq Buffer, 1 × Fluorescence Primer Mixture, 0.3 mM of each dNTP, 0.8 unit Hot-Start Taq DNA polymerase (Takara) and 1 μl of ligation products. After an initial incubation at 95 °C for 2 min, PCR reaction mixture underwent 32 cycles of 20 s at 94 °C, 40 s at 57 °C, 90 s at 72 °C, and a final extension of 60 min at 68 °C. The PCR products were loaded onto ABI 3730xL DNA Analyzer (Applied Biosystems) for sequence analysis.

The repeat locus in the *FMR1* gene was detected with triplet repeat primed polymerase chain reaction as described by Chen and colleagues with minor modifications^52^. This approach included two separate PCR assays: one to amplify the flanking (gene-specific) region and the other to amplify the triplet repeat region. The two sets of PCR products were subjected to capillary electrophoresis for quantifying the number of *FMR1* CGG repeats. Briefly, the PCR assay that amplified the flanking region was performed by preparing a master mix containing a pre-denaturing DNA sample (10 µl), 5 × HotStarTaq PCR buffer (4 µl), 2.5 mM dNTP mixture (1 µl), *FMR1* forward primer (1.5 µl), FAM-labeled *FMR1* reverse primer (1.5 µl), HotStarTaq DNA Polymerase (0.2 µl, Qiagen, Hilden, Germany), and nuclease-free water (1.8 µl). PCR reactions (20 µl) for amplifying triplet repeat region were set up as follows: pre-denaturing DNA template (10 µl), 5 × HotStarTaq PCR buffer (4 µl), 2.5 mM dNTP mixture (1 µl), *FMR1* CGG-RP primer (1.5 µl), VIC-labeled *FMR1* reverse primer (1.5 µl), HotStarTaq DNA Polymerase (0.2 µl, Qiagen, Hilden, Germany), and nuclease-free water (1.8 µl). PCR was conducted on an ABI Veriti™ 96-Well Thermal Cycler (Applied Biosystems) with cycling conditions of 95 °C for 5 min, 35 cycles of (97 °C for 35 s, 63 °C for 30 s, and 66 °C for 4 min), and 60 °C for 60 min followed by a hold at 4 °C. Finally, 1 μl of each PCR product was mixed with 0.5 μl of GeneScan™ 500 LIZ™ dye Size Standard (Applied Biosystems) and 8.5 μl of Hi-Di™ Formamide (Applied Biosystems). The mixture was denatured at 95 °C for 5 min, followed by loading on an ABI 3730xL DNA Analyzer (Applied Biosystems) for capillary electrophoresis.

Multiplex fluorescent PCR, which discriminates 1 bp of difference based on capillary electrophoresis of fluorescently labeled PCR products, was performed to genotype c.3300dupA, c.3637delA, c.3637dupA, c.3870dupA, c.4379delA, and c.4379dupA in the *F8* gene. Roughly, 1 μl of each DNA sample (20 ng/µl) was amplified in a total volume of 10 μl of reaction mixture containing 1 µl of 10 × PCR Buffer (Mg2^+^ plus), 1 µl of dNTP mixture, 1 µl of each FAM-labeled primer (2.5 mM), 0.05 µl Taq^TM^ DNA Polymerase (Takara, Dalian, China), and nuclease-free water (5.95 µl). The mixture was preheated at 95 °C for 5 min, followed by 7 cycles (20 s at 95 °C, 40 s at 64 °C–0.5 °C/cycle, and 1 min at 72 °C), 28 cycles (20 s at 95 °C, 30 s at 60 °C, and 1 min at 72 °C), and a final extension of 30 min at 68 °C. The amplified products were separated with an ABI 3730xL DNA Analyzer (Applied Biosystems) as described above.

All data were visualized in GeneMapper® software v4.0 (Applied Biosystems). The sequences of the specific primers and probes used in this study are presented in Supplementary Table 2.

### Whole gene capture for NGS and data processing

A custom capture kit (Agilent Technologies, Santa Clara, CA, USA) was used to span the whole genomic sequences of ten genes (Supplementary Table 3), and individual targeted capture of each DNA sample was performed according to the manufacture’s recommended instructions. Briefly, 500 ng of purified genomic DNA was enzymatically fragmented to a size range of 150–200 bp, followed by end repair, adaptor ligation, and low-cycle PCR. After purification and size validation, the library was hybridized to the SureSelectXT Custom Capture Library (Agilent Technologies; ID:0770341) for 17 h at 65 °C. The captured hybrids were recovered using streptavidin-conjugated magnetic beads and washed to remove any non-specific bound products. The final libraries were purified and quantitated using quantitative PCR, followed by sequencing on an Ion Proton™ sequencer (Life Technologies, Carlsbad, CA, USA) according to the manufacturer’s recommended protocol. After sequencing, the resulting binary alignment map files were mapped against the human hg19 reference using the Torrent Mapping Alignment Program software, and the Torrent Variant Caller under the default low stringency settings was used to call variants. Variants were annotated using the ANNOVAR software (version 2019Sep29), and allele frequency data were sourced using the dbSNP147, the 1000 Genomes Project, the Exome Aggregation Consortium and the Genome Aggregation Database (gnomAD). The deleterious effects of single-nucleotide variants were predicted by the SIFT, Polyphen-2, M-CAP, CADD, and REVEL programs. The pathogenicity of each variant was classified according to the American College of Medical Genetics and Genomics-Association for Molecular Pathology guidelines.

### Confirmation of variants by alternative methods

All detected variants were confirmed by alternative methods. Roughly, point mutations and small insertions/deletions were confirmed by direct PCR and Sanger sequencing. MLPA kits (MRC-Holland, Amsterdam, The Netherlands) were used to validate larger deletions/duplications in accordance with the manufacturer’s instructions. Large deletions (--SEA, -α3.7, -α4.2, --THAI) in the *HBA* gene were confirmed by Gap-PCR using a commercially available kit (Yilifang Biological, Shenzhen, China) according to the manufacturer’s instructions. The CGG size analysis in for the *FMR1* gene was carried out using the Asuragen AmplideX™ FMR1 PCR Kit (Asuragen; Austin, TX, USA) following the manufacturer’s recommended protocols.

### Reporting summary

Further information on research design is available in the Nature Research Reporting Summary linked to this article.

## Supplementary information

Supplementary Tables

Reporting Summary

## Data Availability

The sequencing data that support the findings of this study have been deposited into the European Variation Archive (EVA; https://www.ebi.ac.uk/eva/) with the accession number PRJEB49380.
