# Publisher Correction: A capillary electrophoresis-based multiplex PCR assay for expanded carrier screening in the eastern Han Chinese population

**DOI:** 10.1038/s41525-022-00282-4

**Published:** 2022-02-21

**Authors:** Ping Hu, Jianxin Tan, Feng Yu, Binbin Shao, Fang Zhang, Jingjing Zhang, Yingchun Lin, Tao Tao, Lili Jiang, Zhengwen Jiang, Zhengfeng Xu

**Affiliations:** 1 Department of Prenatal Diagnosis, State Key Laboratory of Reproductive Medicine, Women’s Hospital of Nanjing Medical University, Nanjing Maternity and Child Health Care Hospital, Nanjing, Jiangsu PR China; 2 Genesky Diagnostics (Suzhou) Inc, Suzhou, Jiangsu PR China

**Keywords:** Molecular medicine, Preventive medicine

Correction to: *npj Genomic Medicine* (2022) **7**:1–9; 10.1038/s41525-021-00280-y, published online 25 January 2022

In the Acknowledgements section of this article the grant number relating to National Key R&D Program of China given for Z.X. and P.H. was incorrectly modified as (No. 2018YFC1002402 to Z.X. and 2021YFC1005300 to P.H.), and should have been (No. 2021YFC1005300 to P.H. and 2018YFC1002402 to Z.X.). The original article has been corrected.

