# Supplementary material for: A capillary electrophoresis-based multiplex PCR assay for expanded carrier screening in the eastern Han Chinese population
Source: NPJ Genom Med. 2022 Jan 25;7:6. doi: 10.1038/s41525-021-00280-y (PMC8789796; doi:10.1038/s41525-021-00280-y)
Supplement: Supplementary file 1 — Supplementary Tables [file 41525_2021_280_MOESM1_ESM.pdf]

Supplementary Table 1 The detailed variants and detection methods of 24 genes

Diseases	Genes	Number of mutations	Variants	Detection methods
Genetic deafness	GJB2 (NM_004004.5)	23	g.20398370-20523823del, c.-23+1G>A, c.9G>A, c.35delG, c.35dupG, c.134G>A, c.139G>T, c.164C>A, c.176_191del, c.230G>A, c.232G>A, c.235delC, c.257C>G, c.283G>A, c.299_300delAT, c.313_326del, c.358_360delGAG, c.416G>A, c.427C>T, c.439G>A, c.508_511dupAACG, c.598G>A, c.560_605dup	iMLDR
	SLC26A4 (NM_000441.1)	60	c.-2071_304+3801del, c.-1374_275del, c.109G>T, c.170C>A, c.227C>T, c.235C>T, c.249G>A, c.269C>T, c.279T>A, c.281C>T, c.416-380_765+519del, c.387delC, c.398C>T, c.414delT, c.439A>G, c.563T>C, c.589G>A, c.716T>A, c.754T>C, c.916dupG, c.919-2A>G, c.946G>T, c.1001+5G>C, c.1079C>T, c.1160C>T, c.1173C>A, c.1174A>T, c.1225C>T, c.1226G>A, c.1229C>T, c.1262A>C, c.1318A>T, c.1327G>C, c.1334T>G, c.1336C>T, c.1340delA, c.1343C>A, c.1343C>T, c.1489G>A, c.1517T>G, c.1520delT, c.1522A>G, c.1540C>T, c.1547dupC, c.1586T>G, c.1594A>C, c.1595G>T, c.1614+1G>A, c.1692dupA, c.1699A>T, c.1707+5G>A, c.1949T>A, c.1975G>C, c.1985G>A, c.1991C>T, c.2027T>A, c.2086C>T, c.2162C>T, c.2167C>G, c.2168A>G	iMLDR
	MTRNR1 (NC_012920)	2	m.1494C>T, m.1555A>G	iMLDR
α-Thalassemia	HBA1/HBA2	5	--SEA, -α3.7, -α4.2, --THAI, Other large segment deletion mutation	HLPA
	HBA1 (NM_000558.3)	3	c.223G>C, c.237delC, c.357_358insTCA	SNaPshot
	HBA2 (NM_000517.4)	9	c.40G>T, c.91_93delGAG, c.95G>A, c.134delC, c.149_150delGC, c.179G>A, c.369C>G, c.377T>C, c.427T>C	SNaPshot
β-Thalassemia	HBB (NM_000518.4)	58	Large segment deletion mutation, c.-140C>T, c.-136C>A, c.-123A>T, c.-100G>A, c.-82C>A, c.-81A>C, c.-80T>C, c.-79A>G, c.-78A>C, c.-78A>G, c.-50A>C, c.-43C>T, c.-12C>T, c.-11_-8delAAAC, c.2T>G, c.25_26delAA, c.27dupG, c.43delC, c.45dupG, c.50dupG, c.52A>T, c.59A>G, c.79G>A, c.85dupC, c.91A>G, c.92+1G>T, c.92+2T>C, c.92+5G>C, c.92+6T>C, c.93-21G>A, c.93-3T>C, c.93-2A>G, c.94delC, c.110delC, c.113G>A, c.115delA, c.125dupT, c.126_129delCTTT, c.126_130delCTTTGinsA, c.130G>T, c.162delT, c.165_177del13, c.216dupT, c.217dupA, c.270_271delTG, c.268_281del14, c.287dupA, c.315+1G>A, c.315+2delT, c.315+5G>C, c.316-197C>T, c.339T>A, c.344T>C, c.364G>T, c.383_385delAGG, c.32A>C, c.108A>C	SNaPshot
Duchenne muscular dystrophy	DMD (NM_004006.2)	1	Large segment deletion and duplication mutation	HLPA
Hemophilia A	F8 (NM_000132.3)	23	Intron 1 inversion, Intron 22 inversion type I & II, Large segment deletion and duplication mutation, c.43C>T, c.977T>C, c.1648C>T, c.1804C>T, c.1903+5G>A, c.3300dupA, c.3637delA, c.3637dupA, c.3870dupA, c.4076G>A, c.4379delA, c.4379dupA, c.5536A>T, c.5879G>A, c.5953C>T, c.6403C>T, c.6506G>A, c.6532C>T, c.6794_6795delAG	SNaPshot, HLPA, AccuCopy and multiplex fluorescent PCR*
Fragile X syndrome	FMR1 (NM_002024.5)	1	CGG repeat mutation (≥55)	Multiplex fluorescent PCR
X-linked ichthyosis	STS (NM_000351.4)	1	Large segment deletion and duplication mutations	HLPA
Spinal muscular atrophy	SMN1 (NM_000344.3)	7	SMN1 deletion mutation, c.22dupA, c.400G>A, c.683T>A, c.689C>T, c.830A>G, c.863G>T	HLPA
Phenylketonuria	PAH (NM_000277.1)	96	c.1-4163_1-406del3758, c.47_48delCT, c.116_118delTCT, c.165T>G, c.168G>T, c.168+5G>C, c.194T>C, c.208_210delTCT, c.284_286delTCA, c.320A>G, c.331C>T, c.440C>T, c.441+1G>A, c.441+2T>A, c.441+3G>C, c.442-1G>A, c.460T>C, c.464G>A, c.466G>C, c.470G>T, c.470G>A, c.472C>T, c.473G>A, c.482T>C, c.494C>A, c.498C>G, c.498C>A, c.509+1G>A, c.526C>T, c.611A>G, c.617A>G, c.671T>C, c.688G>A, c.694C>T, c.707-1G>A, c.716G>A, c.719T>C, c.721C>T, c.722G>A, c.722delG, c.724C>T, c.727C>T, c.728G>A, c.739G>C, c.740G>T, c.754C>T, c.755G>A, c.764T>C, c.770G>T, c.781C>T, c.782G>A, c.827T>A, c.833C>T, c.838G>A, c.839A>G, c.842C>T, c.842+1G>A, c.842+2T>A, c.845A>G, c.856G>A, c.865G>C, c.901C>T, c.907T>C, c.913-7A>G, c.922C>T, c.929C>T, c.935G>T, c.940C>A, c.971T>A, c.975C>G, c.977G>A, c.992T>C, c.1024G>A, c.1033G>A, c.1045T>G, c.1066-11G>A, c.1068C>A, c.1070G>A, c.1084C>A, c.1085C>T, c.1139C>T, c.1162G>A, c.1174T>A, c.1197A>T, c.1199G>C, c.1199G>A, c.1208C>T, c.1222C>T, c.1223G>A, c.1238G>C, c.1243G>A, c.1252A>C, c.1256A>G, c.1301C>A, c.1315+4A>G, c.1315+6T>A	SNaPshot
Tetrahydrobiopterin deficiency	PTS (NM_000317.2)	13	c.58T>C, c.73C>G, c.84-291A>G, c.118_121delTTTG, c.155A>G, c.166G>A, c.186+1G>A, c.200C>T, c.259C>T, c.272A>G, c.286G>A, c.317C>T, c.379C>T	SNaPshot
Methylmalonic acidemia	MUT (NM_000255.3)	25	c.323G>A, c.424A>G, c.494A>G, c.554C>T, c.599T>C, c.682C>T, c.729_730insTT, c.754-1G>A, c.755dupA, c.786T>G, c.914T>C, c.982C>T, c.1061C>T, c.1084-10A>G, c.1106G>A, c.1280G>A, c.1323_1324insA, c.1445-1G>A, c.1630_1631delGGinsTA, c.1677-1G>A, c.1741C>T, c.1880A>G, c.1943G>A, c.2062G>T, c.2080C>T	SNaPshot
Methylmalonic acidemia with homocystinuria Cb1C	MMACHC (NM_015506.2)	19	c.1A>G, c.80A>G, c.81+1G>A, c.146_154del, c.217C>T, c.271dupA, c.315C>G, c.331C>T, c.347T>C, c.365A>T, c.394C>T, c.445_446insA, c.452A>G, c.482G>A, c.567dupT, c.609G>A, c.616C>T, c.626dupT, c.658_660delAAG	SNaPshot
Congenital adrenal hyperplasia	CYP21A2 (NM_000500.7)	23	CYP21A2 deletion mutation, CYP21A2 fusion mutation, Large segment conversion mutation, c.92C>T, c.274A>G, c.292+1G>A, c.293-13A/C>G, c.332_339del, c.449G>C, c.518T>A, c.710T>A;c.713T>A;c.719T>A, c.844G>T, c.923dupT, c.949C>T, c.955C>T, c.1069C>T, c.1225C>T, c.1226G>T, c.1279C>T, c.1450dupC, c.1451G>C, c.1451_1452delGGinsC, c.1455delG	SNaPshot and HLPA#
Hepatolenticular degeneration	ATP7B (NM_000053.3)	70	c.287A>G, c.314C>A, c.525dupA, c.588C>A, c.994G>T, c.1168A>G, c.1470C>A, c.1531C>T, c.1543+1G>T, c.1708-5T>G, c.1708-1G>C, c.1846C>T, c.2078C>G, c.2097_2099delCTT, c.2128G>A, c.2157C>A, c.2294A>G, c.2304dupC, c.2332C>T, c.2332C>G, c.2333G>T, c.2333G>A, c.2337G>A, c.2447+5G>T, c.2464dupA, c.2620G>C, c.2621C>T, c.2662A>C, c.2668G>A, c.2740C>T, c.2755C>G, c.2790_2792delCAT, c.2804C>T, c.2810delT, c.2827G>A, c.2828G>A, c.2906G>A, c.2924C>A, c.2930C>T, c.2939G>A, c.2963G>T, c.2975C>T, c.3029_3030insT, c.3053C>T, c.3074T>G, c.3089G>A, c.3122G>C, c.3140A>T, c.3209C>G, c.3244-2A>G, c.3293C>G, c.3316G>A, c.3426G>C, c.3443T>C, c.3446G>A, c.3452G>A, c.3517G>A, c.3532A>G, c.3646G>A, c.3700delG, c.3744G>C, c.3799G>A, c.3809A>G, c.3818C>T, c.3884C>T, c.3955C>T, c.3982G>A, c.4003G>C, c.4112T>C, c.4114C>T	SNaPshot
Thrombocytopenia-absent radius syndrome	RBM8A (NM_005105)	3	RBM8A deletion mutation, c.-21G>A, c.67+32G>C	HLPA
Hypotonia-cystinuria syndrome	SLC3A1 (NM_000341.3); PREPL (NM_006036.4)	1	SLC3A1/PREPL deletion mutation	HLPA
Xp11.22 microduplication syndrome	HUWE1 (NM_031407.6)	1	HUWE1 duplication mutation	HLPA
Pelizaeus-Merzbacher disease	PLP1 (NM_000533.4)	2	PLP1 deletion and duplication mutations	HLPA
MECP2 duplication syndrome	MECP2 (NM_004992.3)	1	MECP2 duplication mutation	HLPA
1/Int22h2 mediated chromosome Xq28 duplication syı	q28 (154.896Mb-155.335M	1	Int22h1/Int22h2 mediated Xq28 duplication mutation	HLPA

iMLDR, improved multiplex ligation detection reaction; HLPA, high-throughput ligation-dependent probe amplification

*Multiplex fluorescent PCR was used for detecting c.3300dupA, c.3637delA, c.3637dupA, c.3870dupA, c.4379delA, c.4379dupA in F8 gene.

*Human F8 Inversion Mutations Detection Kit based on AccuCopy quantification combined with pre-amplification of long-distance PCR was used for fast analysis of F8 intron inversion.

*HLPA was used for screening the large segment deletion or duplication mutations of F8. The remaining mutations in F8 gene were detected by SNaPshot.

For CYP21A2 gene, point mutations were detected by SNaPshot, and large rearragements were detected by HLPA.

NM accession number is a unique gene identifier that links to the GenBank record.

Supplementary Table 2 Primer sequences used in this study

Primer Name (Locus)	Primer Sequence (5'-3')
ATP7B-E01F	CCTCTCCCGGGACTTTAACACC
ATP7B-E01R	AAAATCCTCCTGGTGGGAGTGAG
ATP7B-E02AF	GCCACCAGCACAGTCAGGATCT
ATP7B-E02AR	CCAGGCTTAAGGGAGCCACTTT
ATP7B-E02BF	GACCCTTCTTGTACCAGCCCAGT
ATP7B-E02BR	CAAGCTCAGGACATGCCTCAAA
ATP7B-E03F	AGTGCTGGGAATTCCATGGTG
ATP7B-E03R	CGAGGTCTATACGCAGCATTCC
ATP7B-E05F	GGCTTTCACAGGCTTTCCTTGAT
ATP7B-E05R	CCATGGGAAAAGTTGAAGAATTTTG
ATP7B-E07F	GTCCTGGACCACAACATCATTCC
ATP7B-E07R	AATATCTGAGGGCCACACACAGC
ATP7B-E08F	TACTTGCTGGCAGCCTTCACTG
ATP7B-E08R	GGAGCAGCTCTTTTCTGAACCTG
ATP7B-E09F	TCATGTCTCTCCAAGCCACAGA
ATP7B-E09R	GTCTCTGCCCACACTCACAAGG
ATP7B-E10/11F	GTGATAAGTGGCGTTTGTTGCAG
ATP7B-E10/11R	TTCCACCAGTTTCACAATCTGAGC
ATP7B-E12F	CCCAATCTTTATCCATGCTTGTGG
ATP7B-E12R	CACAACCACCATATAGCCCAAGG
ATP7B-E13F	TTTCAGAACCCCAACAAGCACAT
ATP7B-E13R	TGGCTCTCAGGCTTTTCTCTCAA
ATP7B-E14F	GGAAGCCCCTCCATCTGTATTG
ATP7B-E14R	GGAGAGAAGGACATGGTGAGGAA
ATP7B-E15F	CACTTCACCCCTCTTGGCTTACA
ATP7B-E15R	CAATACCTTTTTCTGCGGGAAGG
ATP7B-E16F	GGATGCTGTCACAAGAGGTGCTT
ATP7B-E16R	TTTCTGAGAGAGCGGAAGGAAGG
ATP7B-E17F	CCCCAACTTGTGTAGCTGCTGAT
ATP7B-E17R	CTGGGCCAACTGGTGCTTACTTT
ATP7B-E18/19F	CCTTTTGCCAACACTAGGCATTG
ATP7B-E18/19R	CAGCTGGAGCACAGTGGGTAAGA
ATP7B-E20F	CCTCCTCTGTGTCTGTGGTGCT
ATP7B-E20R	ATTGCCTGCTCATGGTGCTGAT
MMACHC-E01F	TAACGGCCCAATTGTCCTTGAG
MMACHC-E01R	AGGAACCCAGGAGGATCAGAGG
MMACHC-E02F	CCTACCGCTGCCAGGACCTA
MMACHC-E02R	TTGGGAGCTGGGGGAAAACT
MMACHC-E03F	AAACTAGGGCTCCCTCGGACAA
MMACHC-E03R	AAGGCTGAGGAATGTGGGACCT
MMACHC-E04F	GGGACCTCCATGACCTTGCTT
MMACHC-E04R	GTTGGGCAGGTGGAGTGGAG
F8-E01F	TCCCCTCCTGGGAGCTAAAGA
F8-E01R	CAGGCAGCTCACCGAGATCA
F8-E07F	AGGCGTCCTTGGAAATCTCG
F8-E07R	AGGGGATCTTGGCTGAGGTCT
F8-E11F2	GAACCCTTGCAACAACAACATGA
F8-E11R	GATGAGGAGAGGGCCAATGAGTC
F8-E12F	CACATGCATGCCATCGCTTT
F8-E12R	TTTCTTTATTCACCACCCACTGGA
F8-E14F	GGAAGGCTTGGGAAATCAAACC
F8-E14R	GGTGCTCGGGGTCAAATGTT
F8-E16F	CAGGCCTCTCGTCCCTATTCC
F8-E16R	AAGCCCAGGCTTTGCAGTCA
F8-E18F2	CCTGGCTTAGTAATGGCTCAGGA
F8-E18R2	GCACAAACAAGCTCATACCTGGA
F8-E22F	CAGGGTGCCCGTCAGAAGTT
F8-E22R	TGTGGAAGCTAAGAGTGTTTGTCCA
F8-E23F2	CGCACAAAGCAAATTAGAAGGAAG
F8-E23R2	cctaCCCATGGTTGAGGGAAGA
F8-E25F	TTGCCCTCAGGTGAATAATCCAA
F8-E25R	TGGCCATCTTGACTGCTGGA
MUT-E02F	AGCCATTCACACGTGGACCATA
MUT-E02R	CGTGCATGTGTTGTTTTCTTTCA
MUT-E03AF	GCTGGTCAGCAGGGATTATCAG
MUT-E03AR	TCTCTTTAGGTACACCTTGTTCTTCTCCA
MUT-E03BF	TGGTACCATCCAAAATGATATACTAAAGGA
MUT-E03BR	GCCCAGATTCCTGCAAGTAACG
MUT-E05F	AAATATAGACCTTGATTTTCTAGTGTGTGATACAA
MUT-E05R	TACCTGCTCAGTAAGTGACCATCCA
MUT-E06F	TGCAGCAGTTGCTGTTTAATCATGT
MUT-E06R	TCTGAAAACAAAGTTGCAAAGTGGA
MUT-E08F	TCCTCTCACACCCCCTTCTCAG
MUT-E08R	TTCAATCTGCCTGTTTCGCACT
MUT-E09F	TCAAATCCAGCAGGGATCAAGC
MUT-E09R	TTTCTTTTGGGCTCACATGGTTT
MUT-E11F	GGTCGCAGACCTCGTCTTCTTG
MUT-E11R	ttgccaagtcagtggctacatacc
MUT-E12F	ACCCTCGCTGCTGGTCATAAAA
MUT-E12R	ATCACCCCTCCACACATGACAA
MUT-E04F2	GTTTATGCTTAGAAAGTGGATTTAATAGGACCTT
MUT-E04R2	TCTGCTAAAGTATAGGCCAGCTCCA
MUT-E10F2	TGCTGTTTATCCATTTGTGTTAAGTGGTG
MUT-E10R2	TGGCCTAAGAAACCTTACATATTACCTCTT
PAH-DELF	gggcacagagctaatatttggaca
PAH-DELR1	caaaggtcagaatttttgccaatc
PAH-DELR2	TAGGCACTTCCGGGACTCTCAG
PAH-E01F	TTGCAAACCTGCCTGTACCTGA
PAH-E01R	GAAACCAGGAAGCACCAGCAGT
PAH-E02F	GCTTGCTTTGTCCATGGAGGTT
PAH-E02R	CATGGAAGTTTGCTACGACATTATCC
PAH-E03F	GGCCTGCGTTAGTTCCAGTGAC
PAH-E03R	TGCTGTTATTTTATGAAGACAGTGTGG
PAH-E04F	CCTGGTTCCCAAGAACCATTCA
PAH-E04R	CCCAGCCCTCGTGTAAATAGGA
PAH-E05F	GCCCCCATTCAAAGCATTCATA
PAH-E05R	TTCTCTCTTCCCCTCAACAAGCAA
PAH-E06F	GTCCCCGACTCCCTCTGCTAAC
PAH-E06R	CCCTCTCCTCTGCCTCAATCCT
PAH-E07F	gcctatgtccctGGGCAGTTATG
PAH-E07R	GTGGACCAGCCAGCAATGAAC
PAH-E08F	TCTGGCTTGGCTTAAACCTCCTC
PAH-E08R	CACCCATTTCAGGTGGGATCAT
PAH-E09F	GTGGGCTGTTCTGAAGGCATCT
PAH-E09R	AACCTGGCTTCCAGGGGAGTAG

PAH-E10F	ccaatcccctccagaaacacc
PAH-E10R	TCCTTGGTTCCTGTGAAGGTCA
PAH-E11F	GGAATCGGGGTGAGATGAGAGA
PAH-E11R	CCCAGAGCTAGTGGCTCACCTT
PAH-E12F	CTCCAAATGGTGCCCTTCACTC
PAH-E12R	ACCCATGGCTTACATGGAGGTG
PTS-E01F	CGTCGCTGCCAGGCACAAGT
PTS-E01R	CCGCTGTACCTGTGTGCACACC
PTS-E02F	GGAACAGAGAAGGGGGTTTGAA
PTS-E02R	CATCCTTATTGAAGGGCTGAAATG
PTS-I01F	AGATAGTAAGCCACTTTGCGGATCA
PTS-I01R	CCCCTTGTTCCACAGCAACTG
PTS-E03F	TGTGCTTGGATGTTGATCTGTTGA
PTS-E03R	GCTGAAAGAGAGCCCAGCAATC
PTS-E04F	TGCAAATGTGGGCACAGTCTCT
PTS-E04R	AGCACCCAACATGCCATTACCT
PTS-E05F	TGGAACAATTTGGAATTTGAGTCGTA
PTS-E05R	ATACAGTGCCACCCACCTCACC
PTS-E06F	ACTTTGATTGTTGTGTGATTTCTGAAGTT
PTS-E06R	CTGGGCTTTGTGCAATGCTAAC
HBA2F	CGCGCCCCAAGCATAAAC
HBA2R	CGGGCAGGAGGAACGGCT
HBBF	CGGCTGTCATCACTTAGACCTCAC
HBBR	GCTGTTTGCAGCCTCACCTTC
HBA1F	CGCGCCCCAAGCATAAAC
HBA1R	GGGGCAAGAAGCATGGCC
CYP21A2SLDF2	CGGGTCGGTGGGAGGGTACCTGAA
CYP21A2SLDR	gatcccaggcgctcgctatgaggt
MUT-E05F	AAATATAGACCTTGATTTTCTAGTGTGTGATACAA
MUT-E05R	TACCTGCTCAGTAAGTGACCATCCA
MUT-E10F2	TGCTGTTTATCCATTTGTGTTAAGTGGTG
MUT-E10R2	TGGCCTAAGAAACCTTACATATTACCTCTT
F8-E12F	GACTGCTAGCTCCTACCTGACAACA
F8-E12R	TTCTTTATTCACCACCCACTGGAC
F8-E01F	TCCCCTCCTGGGAGCTAAAGA
F8-E01R	CAGGCAGCTCACCGAGATCA
MMACHC-E04F-2	CCCTCTCCCCAGCGCATATC
MMACHC-E04R-2	AATCGTTGGGCAGGTGGAGT
MUT-EO6-1F1	TGCAGCAGTTGCTGTTTAATCATGT
MUT-EO6-1R1	TCCAAATACTGCTGCCATTGCT
MUT-EO6-2F1	GGGATTCCCAAAGTGGCTGA
MUT-EO6-2R1	TCTGAAAACAAAGTTGCAAAGTGGA
R20PAR1REF01	TCCCTGTTACATAGTGCTGGGCAGCCTCTGGGGGCCTCACA
R20FAR1REF02	GCCACTGGGGCTTTAGGAATGGTACTTCGCAGCTCAGTGCAGCC
R20VAR1REF03	CAGGCATAACAAACAGCTCAATTCCCACGCATGCAGCCCTCTCCA
R20NAR1REF04	GACAGGAAGGGTGTGGCGCAGACAAATAGCAGCGGTGGTGCC
EXON45_0	ATGGGAAGCCTGAATCTGCGGTGGCAGGAGGTCTGCAAACAGC
EXON67_0	GGATTTTGTGACCAGCGCAGGCTGGGCCTCCTTCTGCATGATT
EXON62_0	GCCTGGCCTACAATACCTGTGTCTTTTTTTTCCTGAGGAAGCTTGG
EXON61_0	TGCATGAAGCCCACAGGGACTTTGGTCCAGCATCTCAGCACTTTC
EXON47_0	GTTTCAGTTACTGGTGGAAGAGTTGCCCCTGCGCCAGGGAATTCTCA
EXON04_0	AGAGTTCATGCCCTGAACAATGTCAACAAGGCACTGCGGGTTTTG
EXON08_0	GCCTCAACAAGTGAGCATTGAAGCCATCCAGGAAGTGGAAATGTTGCC
EXON51_1	CGGTTGAAATCTGCCAGAGCAGGTACCTCCAACATCAAGGAAGATGGC
EXON60_0	TGACTATTGCACACAGGCACTTCGAGGAGAAATTGCGCCTCTGAAAGA
EXON52_1	AGCCTCTTGATTGCTGGTCTTGTTTTTCAAATTTTGGGCAGCGGTAATG
EXON44_0	CCATATGCTTTTACCTGCAGGCGATTTGACAGATCTGTTGAGAAATGGCG
EXON55_1	TACGGGTAGCATCCTGTAGGACATTGGCAGTTGTTTCAGCTTCTGTAAGCC
EXON20_1	GGTGGGTTGGATTTTCAACCAGTTTTCAGCAGTAGTTGTCATCTGCTCCAA
EXON66_0	TGCTTTGATTCTTCATAATAGGGGACGAACAGGGAGGATCCGTGTCCTG
EXON49_1	GCTGAGTGGCTGGTTTTTCCTTGTACAAATGCTGCCCTTTAGACAAAATCTCT
EXON55_0	ACTCATAGATTACTGCAACAGTTCCCCCTGGACCTGGAAAAGTTTCTTGCCT
EXON03_0	TTCAGTTTGGGAAGCAGCATATTGAGAACCTCTTCAGTGACCTACAGGATGGG
EXON49_0	GGGTTCTTTTCCCCAGGAAACTGAAATAGCAGTTCAAGCTAAACAACCGGATG
EXON13_1	TCTGACCTTAAGTTGTTCTTCCAAAGCAGCAGTTGCGTGATCTCCACTAGATTC
EXON10_0	TTGGAAGCTCCTGAAGACAAGTCATTTGGCAGTTCATTGATGGAGAGTGAAGTAA
R20PAR1REF05	GCTGGGGCCTTTTTTCAGCATCCTGGAGCCCTGAACAAGTACAAATG
R20FAR1REF06	GTGCCGGAGAAGATGATTCATGACTCTAGCCCTGCTTTTTCTCTCCCG
R20VAR1REF07	GCAAGTAGGATTGCAAAGGAGGAGGTGAATGGCCAAGTCCTTTTGGAG
R20NAR1REF08	GCTTCCTGGGAGCAGAATTGTTCTTTTTCTTCCCATAGTGTGCCACTGC
EXON12_0	GAATGACTGGCTAACAAAAACAGAAGAAAGAACAAGGAAAATGGAGGAAGAGCCTC
EXON45_1	GAGGATTGCTGAATTATTTCTTCCCCAGTTGCATTCAATGTTCTGACAACAGTTTG
EXON10_1	TGCAATGTGTCCTCAGCAGAAAGAAGCCACGATAATACTTCTTCTAAAGCTGTTTGA
EXON05_0	GATTTGGAATATAATCCTCCACTGGCAGGTAAGAATCCTGATGAATGGTTTCCTTTTG
EXON15_0	TGGCTTTCAGAAAAAGAAGATGCAGTGAACAAGATTCACACAACTGGCTTTAAAGATC
EXON45_2	TCATAGGGAAATTTTCACATGGAGCTTTTGTATTTCTTTCTTTGCCAGTACAACTGCA
EXON18_0	TGATATAACTGAACTTCACAGCTGGATTACTCGCTCAGAAGCTGTGTTGCAGAGTCCTG
EXON07_1	AATTTACCAACCTTCAGGATCGAGTAGTTTCTCTATGCCTAATTGATATCTGGCGATGTT
EXON61_1	CCTGGGAGGAAAAGGAGAGAAATGATGTTCTCTCATTCTATATAATGAGGAACAATTAAAA
EXON14_1	TTAAAAATGGCATGAATAATTTGCCAAAGTATCTCAGTCTCCTATGTACGCTAGAAGTTGGA
EXON15_1	TTTTTATAAGACCATTGAAAGCTAGAAAGTACATACGGCCAGTTTTTGAAGACTTGATAACATTTC
EXON51_0	TGTTACTCTGGTGACACAACCTGTGGTTACTAAGGAAACTGCCATCTCCAAACTAGAA
EXON23_0	TTGAGGGACGCTGGAAGAAGCTCTCCTCCCAGCTGGTTGAGC
EXON69_0	CCATGGTGGAATATTGCACTCCGGTAAGTTTGACGCCAGCCTGACG
EXON17_0	TGCTCAAGAGGAACTTCCACCACCACCTCCCCAAAAGAAGAGGCAGA
EXON43_1	TGGAAATCAAGCTGGGAGAGAGCTTCCTGTAGCTTCACCCTTTCCACA
EXON21_1	CAAAGTCTGCATCCAGGAACATGGGTCCTTGTCCTTTCTCTTTCAGGG
EXON53_1	CATGACTCAAGCTTGGCTCTGGCCTGTCCTAAGACCTGCTCAGCTTCTTC
EXON53_2	GAATCAGTGGGATGAAGTACAAGAACACCTTCAGAACCGGAGGCAACAGTT
EXON27_0	AATGGGAAATGCAAGACTTTGGAAGTCAGTTGCTTTTCTTGGTCTTTGTCA
EXON75_0	GGAAGACCACAATAAACAGCTGGAGTCACAGTTACACAGGCTAAGGCAGCT
EXON22_0	GCAGAGACTCGGGGAATTGCAGGTCTGTGAATATTTGAATGTCAAAACAATAAA
EXON48_1	CCACAGCAGCAGATGATTTAACTGCTCTTCAAGGTCTTCAAGCTTTTTTTCAAG
Y_1_0	CCTACTTGCAGGACTCAATCCGAACTCTGATTCCTTTACCACATAGAGGCTGAG
R20PAR1REF09	CAGCGCTCATTCTTTAGTCTCCGAACAGAATGACAGGGTTTGTGAAGTCG
R20FAR1REF10	TTCATCTGGATCCATGACGATGGACAAGTAGCCTGTCTTCAGTTCCCCTC
R20VAR1REF11	GCCACCCTCCAGTAGCCTTTTCCTGGTGGTTCTGCATTGTACCTGAGAAA
R20NAR1REF12	CCACTGTCCCCTTCATCTGATGATTTCCCCAAAGATGCTGCCTGTAGATG
EXON24_0	CCCTTGGGGATTCAGAAATTCTAAAAAAGCAGCTGAAACAGTGCAGAGTAAGATTT
EXON41_0	GCAAATTTGCTCAGTTTCGAAGACTCAACTTTGCACAAATTGTGAGTTGTTACTGG
EXON59_0	GACTCTCTCCAAGATCACCTCGAGAAAGTCAAGGTACCGTCTACTTCTTTGCTTCA
EXON26_0	GAGATGCACGAATGGATGACACAAGCTGAAGAAGAGTATCTTGAGAGAGATTTTGAA
EXON43_0	GATTTCCAATGGGAAAAAGTTAACAAAATGTACAAGGACCGACAAGGGTAGGTAACA
X_2_0	AACTAGGAATTGGACAAGCCAGTAGGGCCTGGCCTGCTCTAGCTTCTTATATCTACC
EXON17_2	CAACAAGCAAGAACAGTTTCTCATTATTTTCCTTTGCCACTCCAAGCAGTCTTTACTG
EXON70_0	GTCTTAGAGGGGGACAACATGGAAACGTGAGTAGTAGCAAAAGCAGAACACACTCTTG
EXON28_0	CGGAGCTGAGGAAATCTCTGAGGTGCTAGATGTAAGTTGTAAATTAAGCCAAATGATGA

EXON42_0	CAACTTCTCAATGCTCCTGACCTCTGTGCTAAGGACTTTGAAGATCTCTTTAAGCAAGA
EXON72_0	TCACGCATTGAACATTATGCTAGCAGGTATGAGACTAGTTGTATGCCAGGCAAATATTG
EXON48_0	GCTGCTGTGGTTATCTCCTATTAGGAATCAGTTGGAAATTTATAACCAACCAAACCAAGA
EXON36_0	GAGAAAAAGAAACCCCAGCAAAAAGAAGACGTGCTTAAGGTAGCAAATAAAATATGAAAAG
EXON40_0	AAAAAATTAGCCAGCCTACCTGAGCCCAGAGATGAAAGGAAAATAAAGGTAATGTTGTTTT
EXON58_0	TGAGACTGTACGAATATTTCTGACAGAGCAGCCTTTGGAAGGACTAGAGAAACTCTACCAGG
X_3_0	TCTATCTTGCTGTGTGGTGAGAAAAGAAATAATTTAGTGCTTTGCATCTCTCATTCTTCATT
EXON74_0	GAATCCTAGCAGATCTTGAGGAAGAAAACAGGTGAGTTTTCTTTCTAGCTTTGTCATTGGTATG
EXON29_0	GAGACATTTAATTCTCGTTGGAGGGAACTACATGAAGAGGTATGAAGATAAGTGAAAAATCTCTTT
EXON21_2	TGAAACAATAATTTCTGTAAATGGAACCATTCTCCCTACAACCTGTATTAACAAGGAACGAAATACTG
EXON48_2	CAGAGCTTTACCTGAGAAACAAGGAGAAATTGAAGCTCAAATAAAAGACCTTGGGC
EXON02_0	GCATTTTAGATGAAAGAGAAGATGTTCAAAAGAAAACATTCACAAAATGGGTAAATGCACA
EXON77_0	AACTCAACAACTCCTTCCCTAGTTCAAGAGGTAAGCTCCAATACCTAGAAGGGACTCAGATT
EXON46_2	GAACAAAAGAATATCTTGTCAGAATTTCAAAGAGATTTAAATGAATTTGTTTTATGGTTGGAGGAAG
EXON39_0	GGTACTGTAAAAGAATTGTTGCAAAGAGGAGACAACTTACAACAAAGAATCACAGATGAGAGAAA
R20PAR1REF13	TCACACAGAGTCACCCTCCTTCTGAAGTGCTTTGCTTATAGCAGCCTGAACC
R20FAR1REF14	CCTTCCTGCTCATCACTCTTTCCTATGTCTCCATCATGGCCGCCATCT
R20VAR1REF15	GGGATGTGGTGTACACCTACCATCAGTTTATAGAGGCTGCTAAATCGACCTGCG
R20NAR1REF16	GATGAAGGGTCTTTGGCTTCCTTTAGACCAAGAAGGGTAGTGAGATTCTAGCAGCC
R20PAR2REF17	AGATTATTGAAGGTGGCCCAGGCTCAGGGTGGGGGAAGTGGC
R20FAR2REF18	TGCCTAGCTGTGTGACATGATTGGCATCCCCAGTGTGGGACCATG
R20VAR2REF19	CCACCCCAGGAATATCCAATTAGCAAATCATGATCCGGCCCTCCA
R20NAR2REF20	TGGACAGCTTGTCCCACCTCCCTCATGGAGCCCAAGAAGGATTCA
EXON03_1	CAGTTTTTGCCCTGTCAGGCCTTCGAGGAGGTCTAGGAGGCG
EXON11_0	AATTGTTAACTTCCTTCTTTGTCAGGGGTACATGATGGATTTGACAGCCCATCAG
EXON65_0	TTTTTTGCAGTGGATCTCTTGAGCCTGTCAGCTGCATGTGATGCCTT
EXON06_0	TGTAATCAACTTCACCACCAGCTGGTCTGATGGCCTGGCTTTGAATG
EXON54_1	CATCTGCAGAATAATCCCGGAGAAGTTTCAGGGCCAAGTCATTTGCC
EXON07_0	GACTGGAATAGTGTGGTTTGCCAGCAGTCAGCCACACAACGACTGGAA
EXON20_0	CAAGCCTCAGAACAACTGAACAGCCGGTGGATCGAATTCTGCCAGTTG
EXON57_0	TGGTACGCTGCTGTTCTTTTTCAGGTCCCATTTGGAAGCCAGTTCTGA
EXON14_0	GCTGTGCTTGATTGTCTCTTCTCCAGGTATTGGGAGATCGATGGGCAAA
EXON19_0	ACTCATCTTTGCTCTCATGCTGCAGGCCATAGAGCGAGAAAAAGCTGAGA
EXON52_0	CAATGCAGGATTTGGAACAGAGGCGTCCCCAGTTGGAAGAACTCATTACC
EXON09_1	AAATGGGCTCCGTGTAGGGTCAGAGGTGGTGACATAAGCAGCCTGTGTGTA
EXON56_1	AGACAATGAGGAAAATTTGGCCATTTTAATTCATTTGTGGCCTTTTTGCTCC
EXON06_1	AATTACGAGTTGATTGTCGGACCCAGCTCAGGAGAATCTTTTCACTGTTGGT
EXON54_0	AAAATCTATAGCAGTTGGCCAAAGACCTCCGCCAGTGGCAGACAAATGTAGA
EXON57_1	AATGTCCTACCCTATGTACATCGTTCTGCTTCTGAACTGCTGGAAAGTCGCC
EXON04_1	TGGTTTCATTTCTAGTAGATTGTCGGTCTCTCTGCTGGTCAGTGAACACTCTTTTGTTT
EXON09_0	GCACAGGGATATGAGAGAACTTCTTCCCCTAAGCCTCGATTCAAGAGCTATGCC
EXON56_0	CAGCCAAAAAAATCCTGAGATCCCTGGAAGGTTCCGATGATGCAGTCCTG
EXON64_0	AATGTCAGATTCTCAGCTTATAGGACTGCCATGAAACTCCGAAGACTGCAGAAGG
R20PAR2REF21	GCAGCAAAGGCACATTTACTCCGTATTGGAGTTCATCATGCCCAGGG
R20FAR2REF22	TGAGTTCACATCACAGCGAGGGATTCAGGGTATTGAATCTTGTGGGGG
R20VAR2REF23	GGCTGGGAACCTCAAGAGTGAGGCATCCTTGAGCAACTCTGGGTTCTACA
R20NAR2REF24	CTGGAGTGAGGGGAAGAAGCTGTTACAGAAGTGGAATGGTTTCTGGTGG
EXON18_1	ACCTACATTGACTTTTTCTTTTAAGTCTGAGAAGTTGCCTTCCTTCCGAAAGATTGCA
EXON51_2	GCTTGATCAAGTTATAAAATCACAGAGGGTGATGGTGGGTGACCTTGAGGATATCA
EXON11_1	CCTGAGGCATTCCCATCTTGAATTTAGGAGATTCATCTGCTCTTGTACTTCAGTTTC
EXON13_0	AAGAAGATCTAGAACAAGAACAAGTCAGGGTCAATTCTCTCACTCACATGGTGGTGGT
EXON19_1	TAAGAAGATTATCTAAATCAACTCGTGTAATTACCATTCACCATCTGTTCCACCAGGG
EXON47_1	GAGATTTGTCTGCTTGAGCTTATTTTCAAGTTTATCTTGCTCTTCTGGGCTTATGGGA
EXON52_2	ATACATTTTTAAATCAATTCAGGGCTTATATAGTTGCAAAGCATGCATTGATGGGTGTG
EXON44_1	GAGGATTGCTGAATTATTTCTTCCCCAAATATCTTTATATCATAATGAAAACGCCGCC
EXON12_1	CAACATAAGATACACCTACCTTATGTTGTTGTACTTGGCGTTTTAGGTCTTCAAGATCAGGTCC
EXON05_1	CCATCTACGATGTCAGTACTTCCAATATTCACTAAATCAACCTGTTAAAGAAAGGGGTAAAACA
EXON08_1	CATTAAGATGGACTTCTTATCTGGATAGGTGGTATCAACATCTGTAAGCACATTAACACTACACATC
EXON44_2	CAGTTTCTCAGAAAGACACAAATTCCTGAGAATTGGGAACATGCTAAATACAAATGGTATCT
EXON31_0	GGCTGCCCAAAGAGTCCTGTCTCAGATTGATGTTGCACAGGTATATGTTATTTCA
EXON53_0	TCCCTATACAGTAGATGCAATCCAAAAGAAAATCACAGAAACCAAGGTTAGTATCAAAGATACCTTT
EXON73_0	TCTTATCTAAATGATAGCATCTCTCCTAATGAGAGCATGTAAGTATCCCATCTCTTTTTACAAAATGTTCC
Y_2_0	CATGGTTAAAGTGATGGGTTAGCAGGACTATGGGTGAGCTATCTACTTACAGCGAGTG
EXON50_1	TGTATGCTTTTCTGTTAAAGAGGAAGTTAGAAGATCTGAGCTCTGAGTGGAAGGCGG
EXON76_0	CCTATGCTGCTCCGAGTGGTTGGCAGTCAAACTTCGGACTCCATGG
EXON30_0	CAAATGCCTCAGGAAGCCCAGGCAAGTACATCTGGGAATCAGCTTCC
EXON78_0	CCCCTGGAAAGCCAATGAGAGAGGTTAGTGAGATTCAGGCTCACGGCC
EXON67_1	TCCAAGACAGTTGGGTGAAGTTGCATCCTTTGGGGGCAGTAACATTGA
EXON17_1	TCTTGAGCATGCTTTACCAGGATCTGTTCCCTTGTGGTCACCGTAGTTACT
EXON25_0	GTGGGATCACATGTGCCAACAGGTATAGACAATCTCTTTCACTGTGGCTTG
Y_3_0	AGGACACCCCGACATGACTCCTAAAAGTGGCACATAGCTATTTTCAGCCG
EXON26_1	CAGACATGTCTTATTTTCACACTTATGGGAAATTGAGTGTATCTGATCCCCATGAGTTATTTT
EXON21_0	CAGACTTTGTGGCCTTTACAAATCATTTTAAGCAAGTCTTTTCTGATGTGCA
X_1_0	CAGAGCATGCTTCAGTTGCCATGAGAGCCATGAACGGAGAGGTGG
EXON33_0	AAGTCTGAGTGAAGTGAAGTCTGAAGTGGAAATGGTGATAAAGACTGGACGTCAGATTG
R20PAR2REF25	AGAGAGATGGGGGAAGGAAGAGAGACAGGGAAGGAGAGAGCAGGGTTT
R20FAR2REF26	GGCTTTCTTCCCATCCCAGTCAGCCTGAAAAATTGTCTCTATCAGGGAAA
R20VAR2REF27	CCTTTGACTGGTTTGTGGCCATCTGTTGTCCTCTCTGTTACGGCCTCATC
R20NAR2REF28	CCCAATGGTTGCAAGAGGTTCCCTAGGACTAGGGTCTTGCATAGAGCCTTC
EXON02_2	TTTAATTTGGATGCCCCAAACCAGCATCACTCATGTTTAATTCCATTTATCAATG
EXON71_0	TGGCCAGTAGATTCTGCGTGAGTACTTTTTTTGCTGAAGGGTGCTGCTACC
EXON16_1	CAGTTTGCCCATGGATTGCTTTTTCTTTTCTAGATCCGCTTTTAAAACCTGTTAAA
EXON35_0	CAGTTTTGGGCAAGAAGGAGACGTTGGTGGAAGATAAACTCAGTCTTCTGAATAGT
EXON50_0	CCTGGACTGACCACTATTGGAGCCTGTAAGTATACTGGATCCCATTCTCTTTGGCT
EXON46_1	TTAGTTGCTGCTCTTTTCCAGGTTCAAGTGGGATACTAGCAATGTTATCTGCTTCC
EXON63_0	CCCAAAATGACAGAGCTCTACCAGTCTTTAGGTAAGGACATGGCCATGTTTCCTCC
EXON38_0	CAGCAGGGGGTGAATCTGAAAGAGGAAGACTTCAATAAAGATATGGTAAATTGGTTG
EXON01_1	AAAAAGTAACACTTCAGTTTTTCCTATTCGTTTTTCTCCGAAGGTAATTGCCTCCCAG
EXON79_0	TTTCTCTTTGTTTTCCAGGACACAATGTAGGAAGTCTTTTCCACATGGCAGATGATT
EXON16_0	GCTGGATAACTTTGCCCGGTGTTGGGATAATTTAGTCCAAAAACTTGAAAAGAGTACA
EXON68_0	TGTAACATCTGCAAAGAGTGTCCAATCATTGGATTCAGGTATTAGGAACCAAAAAAAA
EXON50_2	GGAGAAAGGGTTTTTGTATGGAGCAATTGATAAATATTTGTAGGGTGGTTGGCTAAAA
EXON34_0	GGATTCTGAAGTTGCCTGGGGAAAGGTAAAACCTATATCACTGAAGGTTATTTTGAACA
EXON43_2	GAGCGATCCACTCTCTCAGGATGAGGTCTGTGATTTACCTATTGAATTTGAACATGTCA
EXON46_0	GCAACTAAAAGAAAAGCTTGAGCAAGTCAAGGTAATTTTATTTTCTCAAATCCCCCAGGG
EXON01_0	GCCTACTGGAGCAATAAAGTTTGAAGAACTTTTACCAGGTTTTTTTTATCGCTGCCTTG
EXON16_2	GCGGGCACGATTGAAATATTTTCTTATCTATGCAAATGAGCAAATACACGCA
EXON37_0	GCCATTTCACACAGAATTAAGACTGGAAAGGTAGGAAGATCTACTCCAAGGTGGAAACTTG
EXON32_0	AAAATTAAATCAGTGCCTTTTTACACTGTCCTTACAGAAAAAATTACAAGATGTCTCCATGAAGTTT
EXON02_1	TCTTAAAAATCCGTTTTATTTCTCTCATAGTGTCATTTTATTTTAGCTGTGCAAATCTGGAAATATTATG
EXON65_1	CACTATTTATGACCGCCTGGAGCAAGAGCACAACAATTTGGTCAACGTCC
R20PAR2REF29	CACTGTGATTCAGCAGTGAAGTCCTGCTCTCTCGAGCTAACATTGTGGAGGG
R20FAR2REF30	AAGGCCAGGCCTGTATTTAGTTAGTGGTAATGATCTCGTTAGGGGTGTGAATGG
R20VAR2REF31	CGTACAGACTTTAGGGAGCCTGTGTTCAGTGTGGCCTATAAGGATTTGGGTATGG
R20NAR2REF32	GGGTGATAGTGGAGAGTCTTACCTTCCACACCAGATCCAGGAGACTGTTAGGTCAC
EXON61_2	TAGAAGCATAAATCATCCTGAAATATAACTTGGTTGATAAGTTGAGAAGGTTAGGAAGAGGAAGGCC
EXON61_3	GAGTTATGAAATGTTGCTTTAGTGTTCTCAGTCTTGGAGACTCAAAGTATCTTATCAGAAAGG

EXON03_2	CTTCATCCGTCATCTTCGGCAGATTAATTATGCACACTAATTATCCTTAAATATAGCT
R20FAR1REF02	GCCACTGGGGCTTTAGGAATGGTACTTCGCAGCTCAGTGCAGCC
CYP21A1PUS2C	CGGTGGGAAGGCACCTGAGGGTGGGGTCAAGGGAGGC
CYP21A2US2D	TCGGTGGGAGGGTACCTGAAGGTGGGGTCAAGGGAGGC
CYP21A2DS3C	cacaggcttcctcaccacgggtgagatggactgggacccg
R20FAR1REF06	GTGCCGGAGAAGATGATTCATGACTCTAGCCCTGCTTTTTCTCTCCCG
CYP121A2-E06	AGGGAGAGGCTCCTTCCCACAGCTGCATTCTCATGCTTCCTGC
CYP121A2-E08	CCTACAAGGACCGTGCACGGCTGCCCTTGCTCAATGCCACCAT
CYP121A2-E10-1	CTTCGTGGTGCTGACCCGACTGCTGCAGGCCTTCACGCTGCTG
R20NAR2REF24	CTGGAGTGAGGGGAAGAAGCTGTTACAGAAGTGGAATGGTTTCTGGTGG
C4A-3	GCAGATCGTGTTCATGAATCGAGAGCCCAAGAGGACCCTGACCTC
CYP21A2-E01-E02	GGTCCTCTCTCCGCTGACGCTGCTTTGGCTGTCTCCCAGATGT
R20FAR1REF10	TTCATCTGGATCCATGACGATGGACAAGTAGCCTGTCTTCAGTTCCCCTC
CYP121A2-E07	CAGGCCAGTGGAGGGACATGATGGACTACATGCTCCAAGGGGTG
CYP21A2E05-2	TGATTCCCTTTCTCAGGGTGAGGACCTGGAGCCTAGACACCCCTG
CYP21A2-E04	CCTGTGGCCATTGAGGAGGAATTCTCTCTCCTCACCTGCAGCATCA
R20VAR2REF23	GGCTGGGAACCTCAAGAGTGAGGCATCCTTGAGCAACTCTGGGTTCTACA
CYP121A2-E09-2	GAGACGGTCTGGGAGAGGCCACATGAGTTCTGGCCTGGTATGTGG
CYP121A2-E10-3	GAGCGAAAGTTTCTTGGTCTCAGCTTCATTTCCGTGAAGGGCACCGAGAA
R20FAR1REF15	GGGATGTGGTGTACACCTACCATCAGTTTATAGAGGCTGCTAAATCGACCTGCG
R20FAR2REF18	TGCCTAGCTGTGTGACATGATTGGCATCCCCAGTGTGGGACCATG
CYP21A2DS2	CTCACTCTCCTCAAAGCCTCGGACCGAGACCACGGTCACCTCATA
CYP21A2-E01	AGGGCCCTGAGGTGCCACTTATAGCTCAAGAGCCCCAGCCATCCCT
C4A-4	GTGGTGGCTCGAGGGTCCTTCGAATTCCCTGTGGGAGATGC
R20FAR2REF22	TGAGTTCACATCACAGCGAGGGATTCAGGGTATTGAATCTTGTGGGGG
CYP21A2-E02	GATGTGGTGGTGCTGAACTCCAAGAGGACCATTGAGGAAGCCATGGT
C4A-1	TGGAGGTCAGTAATGCCCATATTCAGCTTCTCCAGGGCGTCCTGGAACT
CYP21A2E03S	GACCTGTCCTTGGGAGACTACTCCCTGCTCTGGAAAGCCCACAA
R20VAR2REF27	CCTTTGACTGGTTTGTGGCCATCTGTTGTCCTCTCTGTTACGGCCTCATC
CYP21A2E06S	CCATAGAGAAGAGGGATCACATCGTGGAGATGCAGCTGAGGCAGC
C4A-6	GTGTTCATGAAAGCGGACACAGCAGTGCTTCCAGCTTCATGGTTCC
CYP21A2-E03	AGTGGTGGAGCAGCTGACCCAGGAGTTCTGTGAGGTAAGGCTGGGC
R20FAR2REF26	GGCTTTCTTCCCATCCCAGTCAGCCTGAAAAATTGTCTCTATCAGGGAAA
CYP21A2E05-1	GTATCCAGGAGGTGTTAAAAACCTGGAGCCACTGGTCCATCCAAATTGTG
CYP121A2-E09	CTACGACATCCCTGAGGGCACAGTCATCATTCCGAACCTCCAAGGC
CYP21A2-E04-2	AGGGGCTGTGAGGCACCTTGATCTTGTCTCCGAAGGTGAGGTAACAG
R20PAR1REF13	TCACACAGAGTCACCCTCCTTCTGAAGTGCTTTGCTTATAGCAGCCTGAACC
R20FAR2REF30	AAGGCCAGGCCTGTATTTAGTTAGTGGTAATGATCTCGTTAGGGGTGTGAATGG
CYP21AP01	TGATGTGGAACCAGAAAGCTGACTCTGGATGCAGGAAAAAGGTCAGG
CYP21A1PDSX	TTTTCTTGgCTCCCACCTGGAGTTTCTGGGTCCGGGCCCG
CYP21A1PDSX	TTTTCTTGgCTCCCACCTGGAGTTTCTGGGTCCGGGCCCG
CYP21ATE500	GAGATGTTCCCAAAACCATGGGCATAGTCCTCCCAGTCCCTCCA
R20PAR1REF01	TTCCCTGTTACATAGTGCTGGGCAGCCTCTGGGGGCCTCACA
R20FAR1REF02	GCCACTGGGGCTTTAGGAATGGTACTTCGCAGCTCAGTGCAGCC
R20VAR1REF03	CAGGCATAACAAACAGCTCAATTCCCACGCATGCAGCCCTCTCCA
R20NAR1REF04	CCAAGAGGGAAGGCAGGCAGAAGACAAATAGCAGCGGTGGTGCC
SMN1E8.5FG	GAAAAACCATCTGTAAAAGACAGGGGTGGGGGTGGGAGGCCAGCA
SMN2E8.5FA	GAAAAACCATCTGTAAAAGACAGAGGTGGGGGTGGGAGGCCAGCA
SMNc.22dupA|W	CCGGGACGCCGCCACCACTGCCGCCGCTGCTCATCGCC
SMNc.22dupA|M	CGGGACGCCGCCACCACTTGCCGCCGCTGCTCATCGCC
STSE05-2	CAGGGGCCGGAAGTAATGAAGGAAGCCCAAGAAAAGGGTCA
STSE06-1	CCTACCTCCACGTGCACACAGCCCTGTTCTCCAGCAAAGAC
STSE09-1	TGATCTGATGCCCCTGCTTGAAGGAAAAAGCCAACGCTCCG
F8E01-2	ACATGCCTTTACCTTGCGTCCACAGGCAGCTCACCGAGATCA
STSE06-2	GCAAAGACTTAGCAGCTTTCTAAGCACTCACTGAGGAGAAACGTACCCACA
F8E14-2	CGACATATCTGGATTTTGTGCATCTGGTGGAATGGGGCCCTCTTT
STSE04-2	CCTCCCTCCCATTCCCATACCTATAGATGGTTCCCATCCCATAGATG
R20PAR1REF05	GCTGGGGCCTTTTTTCAGCATCCTGGAGCCCTGAACAAGTACAAATG
R20FAR1REF06	GTGCCGGAGAAGATGATTCATGACTCTAGCCCTGCTTTTTCTCTCCCG
R20VAR1REF07	GCAAGTAGGATTGCAAAGGAGGAGGTGAATGGCCAAGTCCTTTTGGAG
R20NAR1REF08	GCTTCCTGGGAGCAGAATTGTTCTTTTTCTTCCCATAGTGTGCCACTGC
STSE01-1	CCCAAGCACAGTGCTGTTGGCCAAGCCTCCAGCAGCTGAC
STSP03-1	CACAGGAAGTTCACATCATCACCGTCAGCTTCTTTGTGGGCACCA
F8E02-2	CATTGTTTTCATTACCCATCCAGGGTGGCCTTGGCTTAGCGATGTT
F8E20-1	GTGTTCCACCCGTTTCATTTCAGAGTGTCAGACTCCCCTGGGAATG
STSE02-2	CAACAATAACCATCCCGGGGACTGTTGCCTATGAATACAGCCATGT
STSE03-1	TTTGTTCTAGGACTCCCAATATCGTCCGGTTGGCCAGTGGGGGAGT
STSE07-1	GTCTTTTTCCCCTCCAGGGCAGATCTTGAACCTTCTGGATGAGCTG
STSE10-5	TCATGACAACATGAAACCTATTGGGACAGCATGACTGTGCAGGGTC
F8E03-1	GGCGGACATCTCATTCTTACAGGTCTGCTAGGTCCTACCATCCAGGCT
F8E14-1	AGAGTCTTATTCTTCCTCATCTCCAGGTCTATGGATTCTGGGGTGCCA
STSE01-2	ATCCTCTGTTCATGGTTTCTGCACCAGTGGGAGTTTAATAAGCATATTCC
STSE02-1	GGAAGATGAAGATCCCTTTCCTCCTACTGTTCTTTCTGTGGGAAGCCG
SMNc.400G>A|W	tgtgttgtggtttacactggatatggaaataCaGaggagcaaaatctgtccgatctactttc
F8E17-1	AGGCCTGATTGGACCCCTTCTGGTCTGCCACACTAACACACTGA
F8E05-1	TGTTCTCACTTCTTTTTCAGGGAGTCTGGCCAAGGAAAAGACACAGACC
SMNc.400G>A|M	tgtgttgtggtttacactggatatggaaataCaAaggagcaaaatctgtccgatctactttc
F8E11-1	GCTTGTGGGTAGGTGTAAAACATTTGAAGGATTTTCCAATTCTGCCAGG
R20PAR1REF09	CAGCGCTCATTCTTTAGTCTCCGAACAGAATGACAGGGTTTGTGAAGTCG
R20FAR1REF10	TTCATCTGGATCCATGACGATGGACAAGTAGCCTGTCTTCAGTTCCCCTC
R20VAR1REF11	GCCACCCTCCAGTAGCCTTTTCCTGGTGGTTCTGCATTGTACCTGAGAAA
R20NAR1REF12	CCACTGTCCCCTTCATCTGATGATTTCCCCAAAGATGCTGCCTGTAGATG
F8E22-2	ACCATTAAGGTTCCAGTGGAATTTCCTCGATAAGTCTGCCACTTCTTCCC
F8E24-2	AGTAAATCTGTTGCCTCTTACCTGAGGTCTCCAGGCATTACTCCTCCCTTG
F8P01-1	CATGCTCCACATTAAGTGACCAATTTCACAGAAGCCAATGTGTTTTCAC
F8E01-1	AAAGGAAGCAATCCTATCGGTTACTGCTTAGTGCTGAGCACATCCAGTGG
SMN1E7	tttttttaacttcctttattttccttacagggtGtcagacaaaatcaaaaagaaggaaCgtgct
SMNE09-1	CTCATTTGCAGGAAATGCTGGCATAGAGCAGCACTAAATGACACCACTAAAGAA
F8E13-2	ATAACTAACCTGGGTTTTCCATCGACATGAAGACAGTTTCTCCTGAGAATGG
F8E18-2	CAGTGAACACATGTCCACTGAAATGAATAGAATGGATGTTTTCATTGCTGCC
SMN2E7	tttttttaacttcctttattttccttacagggtGttagacaaaatcaaaaagaaggaaCgtgct
SMNE05-1	GCCAAGTTTCAACAGATGAAAGTGAGAACTCCAGGTCTCCTGGAAATAAAT
F8E09-2	TCTTCAACTTACCAACAGTGTGTCTCCAACTTCCCCATAAAGTAAAGGTCCCA
F8E18-1	TCTCTGTGTCCTTCTCCAGCAATCAATGGCTACATAATGGATACACTACCTGG
F8E23-2	CATCCGTTTGCACCCAACTCATTATAGCATTCGCAGCACTCTTCGC
SMNc.830A>G|W	catggtacatgagtggctatcatactggctaGtAtatggtaagtaatcactcagcatcttttcctg
F8P03-1	CAGACGCCCATTTAGCAGAACAAAATATGGATGTCTGAGTACCTGGGTACTCCA
F8E07-2	TTACCATGTTGGTGGGAAGAGATATGACAAAACAGTAGAAACTGTCCAAGGTCC
STSE04-1	CCACCGATGAGATTACCTTTGCTAAGCTTCTGAAGGATCAAGGTTATTCAAC
SMNc.830A>G|M	catggtacatgagtggctatcatactggctaGtGtatggtaagtaatcactcagcatcttttcctg
SMNE02-1	TTATTTCTTACCCTTTCCAGAGCGATGATTCTGACATTTGGGATGATACAGC
STSP04-1	CGTGTGTAAAAACTGTATCCATCTCCAAGCTCAGGAAAGGAACAATTTACAGTC
R20PAR1REF13	TCACACAGAGTCACCCTCCTTCTGAAGTGCTTTGCTTATAGCAGCCTGAACC
R20FAR1REF14	GGCTTCTCAGTTCTAAGCCTCACAAATCCTTTCTACCTCCCACAGTGTTTGGG
R20VAR1REF15	GGGATGTGGTGTACACCTACCATCAGTTTATAGAGGCTGCTAAATCGACCTGCG
R20NAR1REF16	GATGAAGGGTCTTTGGCTTCCTTTAGACCAAGAAGGGTAGTGAGATTCTAGCAGCC
F8E26-2	TTGCCCTGGGTTGCAATCTTTGGATGCTTCCTCCAACTGC

<table>
<tr><td>STSE10-2</td><td>GCTGCTAGCGGCTCAGTCTCTTATCCTGTTTTTCTCTATCACACTGGC</td></tr>
<tr><td>R20PAR2REF17</td><td>AGATTATTGAAGGTGGCCCAGGCTCAGGGTGGGGGAAGTGGC</td></tr>
<tr><td>R20FAR2REF18</td><td>TGCCTAGCTGTGTGACATGATTGGCATCCCCAGTGTGGGACCATG</td></tr>
<tr><td>R20VAR2REF19</td><td>CCACCCCAGGAATATCCAATTAGCAAATCATGATCCGGCCCTCCA</td></tr>
<tr><td>R20NAR2REF20</td><td>TGGACAGCTTGTCCCACCTCCCTCATGGAGCCCAAGAAGGATTCA</td></tr>
<tr><td>STSE05-1</td><td>TGAGAGACTGCAAGCCCGGAGAGGGCAGTGTCTTCACCACG</td></tr>
<tr><td>STSE03-2</td><td>GGAGATACCTCGAGGTGGGAGACTGTCCCGAAGGTGACCACA</td></tr>
<tr><td>STSE10-1</td><td>AGGGGCATTCACAAGGCACACCAGTGCAAGCAGATGACAAAA</td></tr>
<tr><td>F8E21-1</td><td>CTAACCCAGCTGAATTTAATCTCTGTTTCTTTACTTGGGCAAAGGACAGTGG</td></tr>
<tr><td>STSE10-3</td><td>CACACAGCGGTTTGCAGAGAATGTTAGCCATGACTTGGGCTTT</td></tr>
<tr><td>F8E08-2</td><td>GGGCGAGGACTAAGGGAGCATAGTCCCAGTCCTCCTCTTCAGCA</td></tr>
<tr><td>STSE08-1</td><td>GGAAAAGCAAACAACTGGGAAGGAGGTATCCGGGTTCCAGGCAT</td></tr>
<tr><td>SMNc.683T>A|M</td><td>ATGGAGGCAGCCAGCATGATACTTAGTGGGGTGGTGGTGGTGGC</td></tr>
<tr><td>F8E15-1</td><td>CCTCTGCTTTTTTTCTCCAGGGCTCAGAGTGGCAGTGTCCCTCAGT</td></tr>
<tr><td>F8E19-1</td><td>TTGAGACAGTGGAAATGTTACCATCCAAAGCTGGAATTTGGCGGGTG</td></tr>
<tr><td>SMNc.683T>A|W</td><td>ATGGAGGCAGCCAGCATGATACTAAGTGGGGTGGTGGTGGTGGC</td></tr>
<tr><td>R20PAR2REF21</td><td>GCAGCAAAGGCACATTTACTCCGTATTGGAGTTCATCATGCCCAGGG</td></tr>
<tr><td>R20FAR2REF22</td><td>TGAGTTCACATCACAGCGAGGGATTCAGGGTATTGAATCTTGTGGGGG</td></tr>
<tr><td>R20VAR2REF23</td><td>GGCTGGGAACCTCAAGAGTGAGGCATCCTTGAGCAACTCTGGGTTCTACA</td></tr>
<tr><td>R20NAR2REF24</td><td>CTGGAGTGAGGGGAAGAAGCTGTTACAGAAGTGGAATGGTTTCTGGTGG</td></tr>
<tr><td>F8E10-2</td><td>ttgaggtccggccaacagctggagaaaggaccaacataattttagttg</td></tr>
<tr><td>F8E17-2</td><td>CTTTAAAAGTGGGATCTTCCATCTGGATATTGCAGGGAGCCCTGCAG</td></tr>
<tr><td>F8E21-2</td><td>TTCTAACCTTGATCCAAGAAAAGGGCTCCTTGGTGCTCCAGGCATTG</td></tr>
<tr><td>F8E10-1</td><td>AGCAAGCAGACCATATAACATCTACCCTCACGGAATCACTGATGTCCGT</td></tr>
<tr><td>F8E12-2</td><td>ACTGGACTTAAGTGCTGCTTTACTCACTGTGCATGATGTTGGAGGCTTG</td></tr>
<tr><td>F8E24-1</td><td>CTTTGAGGTTGCAGCATGCCATTGGGAATGGAGAGTAAAGCAATATCAG</td></tr>
<tr><td>STSP02-1</td><td>AATCTATTGGCTGGATGGTCCAGTCCCAAAACAGAAAGAAGAAAGGG</td></tr>
<tr><td>STSE07-2</td><td>CCATGAATTTCTCCTTTGGAAGACACTTCTTCTACATGTGCTCCCTGGT</td></tr>
<tr><td>F8P02-1</td><td>agaggtacacaaaagttattcaggccgacaagaaatgatgccacatggaa</td></tr>
<tr><td>F8E09-1</td><td>AAGTACAAAAAAGTCCGATTTATGGCATACACAGATGAAACCTTTAAGACTCGTG</td></tr>
<tr><td>F8E22-1</td><td>TTAATTGGTAGGTGGATCTGTTGGCACCAATGATTATTCACGGCATCAAG</td></tr>
<tr><td>F8E08-1</td><td>ACTGATTCTGAAATGGATGTGGTCAGGTTTGATGATGACAACTCTCCTTCC</td></tr>
<tr><td>F8E26-1</td><td>CTTTCCTCTTTCAGGTTTTTCAGGGAAATCAAGACTCCTTCACACCTGTGG</td></tr>
<tr><td>STSE08-2</td><td>AAGGGAGCTCCAGCCAGCTTGGCTACTGTAGGAAATATGTCCATGTTG</td></tr>
<tr><td>STSE09-2</td><td>AGGTCTGTTTACTCACTGTTCTGAGGGTGCCAGCGCACAGCATTTAAG</td></tr>
<tr><td>F8E06-1</td><td>CTTTGCCTTCTCTCTCATCAGGGAAAAGTTGGCACTCAGAAACAAAGAACTC</td></tr>
<tr><td>F8E13-1</td><td>GTTTGCAGTTGTCAGTTTGTTTGCATGAGGTGGCATACTGGTACATTCTAAG</td></tr>
<tr><td>F8E16-2</td><td>GTCAGCACAATAGACACCTGCTTACCAGGTCAACATCAGAGAAATAAGCCCA</td></tr>
<tr><td>F8E25-1</td><td>TACTAGTCCAACTCTATTGCCCTCAGGTGAATAATCCAAAAGAGTGGCTGCA</td></tr>
<tr><td>F8E19-2</td><td>TTGCTACTCACTATTGCTGTACACCAGAAAAAGTGTGCTCATCCCAGCA</td></tr>
<tr><td>F8E02-1</td><td>CGACTTTTTCCTTTCTTCACGCAGATTTCCTCCTAGAGTGCCAAAATCTTTTC</td></tr>
<tr><td>F8E14-3</td><td>GCAACAGAAAGCTCTGCAAAGACTCCCTCCAAGCTATTGGATCCTCTTGCTTG</td></tr>
<tr><td>F8E23-1</td><td>GTGGATTCATCTGGGATAAAACACAATATTTTTAACCCTCCAATTATTGCTCG</td></tr>
<tr><td>R20PAR2REF25</td><td>AGAGAGATGGGGGAAGGAAGAGAGACAGGGAAGGAGAGAGCAGGGTTT</td></tr>
<tr><td>R20FAR2REF26</td><td>GGCTTTCTTCCCATCCCAGTCAGCCTGAAAAATTGTCTCTATCAGGGAAA</td></tr>
<tr><td>R20VAR2REF27</td><td>CCTTTGACTGGTTTGTGGCCATCTGTTGTCCTCTCTGTTACGGCCTCATC</td></tr>
<tr><td>R20NAR2REF28</td><td>CCCAATGGTTGCAAGAGGTTCCCTAGGACTAGGGTCTTGCATAGAGCCTTC</td></tr>
<tr><td>STSE10-4</td><td>TCAGATTTTTCTGAAGGGCATACAATGAAAAGTGAAGGGGAAACACACACAC</td></tr>
<tr><td>STSP05-1</td><td>GCAAACATATTGGAATGTGTTGCTCCTCCAAGCTGGTCAGAGACATTTGC</td></tr>
<tr><td>F8E15-2</td><td>CAACTTCTGCTCTTATATATGGCCCCAGGAGTCCCAAATGTTCATTTAGTTCTC</td></tr>
<tr><td>STSP01-1</td><td>TCATTCCAAACCAACATAGAGCTGTGTGATCCAAGGGCCTTAAATAAGAGG</td></tr>
<tr><td>F8E03-2</td><td>GACAATAGGAGGGTATTTTACTCACCCTCAGAAGCTTTCCAGTAGGATACACCA</td></tr>
<tr><td>F8E12-1</td><td>GTCAGACAAGAGGAATGTCATCCTGTTTTCTGTATTTGATGAGAACCGAAGCTG</td></tr>
<tr><td>SMNc.863G>T|M</td><td>AGACTTACTCCTTAATTTAAGGAATGTGAGCTCATTCCTTCTTTTTGATTTTGTCTGAAACCC</td></tr>
<tr><td>F8E04-1</td><td>GACCAGTCAAAGGGAGAAAGAAGATGATAAAGTCTTCCCTGGTGGAAGCC</td></tr>
<tr><td>F8E06-2</td><td>GGATTGTTGAGCAGGTGTGTACATACCTGGCAGAGACCTGTTTACATAACCATTG</td></tr>
<tr><td>F8E20-2</td><td>TGTATTTGAGAGGCACTTATGGAATAGAACTAATAGAAGCATGGAGATGGATTCA</td></tr>
<tr><td>SMNc.863G>T|W</td><td>CAGACTTACTCCTTAATTTAAGGAATGTGAGCTCCTTCCTTCTTTTTGATTTTGTCTGAAACCC</td></tr>
<tr><td>F8P04-1</td><td>TGAGTGACCGTGTTTGTTCATGTAAACATTGAACAAATATTTATCGGCTTCTGCG</td></tr>
<tr><td>F8E07-1</td><td>CTACTTTACAGGTCTGATTGGATGCCACAGGAAATCAGTCTATTGGCATGTG</td></tr>
<tr><td>F8E16-1</td><td>CTACAGGTAACTTTCAGAAATCAGGCCTCTCGTCCCTATTCCTTCTATTCTAGCC</td></tr>
<tr><td>F8E25-2</td><td>tctttcCAAGGAGACCAGCTTACCTTTACTTTGCCATTCTGAAAAAAGAGAGTCC</td></tr>
<tr><td>F8E11-2</td><td>AGAACTCACCTGGTTTCCTCTTTGATCTACAGATTCTTTGTAGCAGATGAGGAGAG</td></tr>
<tr><td>F8E05-2</td><td>TCAGGAATCCAAAATTCAGATTAAGACTCACTAACCTTCATCAAATACAGCAAAAA</td></tr>
<tr><td>F8E04-2</td><td>ACATACTAGTAGGGCTCCAATGAGGCCTGAATTCAAGTCTTTTACCAGGTCCACA</td></tr>
<tr><td>SMNE03-2</td><td>ACCTGTTGTAAGGAAGCTGCAGTATTCTTCTTTTGGCTTTTATTCTTCTTAGCAGG</td></tr>
<tr><td>R20PAR2REF29</td><td>CACTGTGATTCAGCAGTGAAGTCCTGCTCTCTCGAGCTAACATTGTGGAGGG</td></tr>
<tr><td>R20FAR2REF30</td><td>AAGGCCAGGCCTGTATTTAGTTAGTGGTAATGATCTCGTTAGGGGTGTGAATGG</td></tr>
<tr><td>R20VAR2REF31</td><td>CGTACAGACTTTAGGGAGCCTGTGTTCAGTGTGGCCTATAAGGATTTGGGTATGG</td></tr>
<tr><td>R20NAR2REF32</td><td>GGGTGATAGTGGAGAGTCTTACCTTCCACACCAGATCCAGGAGACTGTTAGGTCAC</td></tr>
<tr><td>HBA2DS01</td><td>AGGGCCCGTTGGGAGGCCCAGCGGGCAGGAGGAACGGCTA</td></tr>
<tr><td>HBAE02A</td><td>TCTGCTTCTCCCCGCAGGATGTTCCTGTCCTTCCCCACCACC</td></tr>
<tr><td>HBATHAIJunc</td><td>TATAATTCAGGCCGGGCGCGGCTCAAGGGCTCAGCCCA</td></tr>
<tr><td>HBZD</td><td>ACCGCGTAATGCGCCAATAAACCAATGAACGAAGCAGCG</td></tr>
<tr><td>R20PBR1REF01</td><td>GACAGGAAGGGTGTGGCGCTCACTTGGGAAGCACAGCTCTGG</td></tr>
<tr><td>R20FBR1REF02</td><td>GCCAGCCTTAGCCAAATGCAGCTCTGGTGGCCTTGAAAAACAACC</td></tr>
<tr><td>R20VBR1REF03</td><td>CTCAGTGCTCATAAACCTGTGGCCACTGGGCTCAATGCCACTTGC</td></tr>
<tr><td>R20NBR1REF04</td><td>TGTTTTGCATTCCTCTTCCTGGCAGTGGTCAGGTGGCTTCCAGC</td></tr>
<tr><td>HBA1DS01</td><td>GGAGCCTCGGTGGCCATGCTTCTTGCCCCTTGGGCCTCC</td></tr>
<tr><td>HBA1DS02</td><td>TCAGCAAACGTGCCAGGCATGGGCGTGGACAGCAGCTGG</td></tr>
<tr><td>HBQ1E01</td><td>CCCTGTGGAAGAAGCTGGGCAGCAACGTCGGCGTCTACA</td></tr>
<tr><td>HBQ1E03</td><td>CGCCGAGATAACGTGGCTCAGGAACTTGTCCAGCGACGC</td></tr>
<tr><td>HBASEAJunc</td><td>gcgccttggggaggttcacttggaggctggggcaggag</td></tr>
<tr><td>HBAE01B</td><td>GTTGGTCTTGTCGGCAGGagACAGCACCATGGTGGGTTCt</td></tr>
<tr><td>HCS-6</td><td>CTGAGTCAGGGTCGCGCCTCCGTTGGAAACTTGGGCTGAG</td></tr>
<tr><td>RBM8A_SNP2|G</td><td>GAGACGGCTGGTGGGAAACGGGGAAGGTGCGAGAGAAGGC</td></tr>
<tr><td>HBAE02B</td><td>CCGCTCACCTTGAAGTTGACCGGGTCCACCCGAAGCTTGTG</td></tr>
<tr><td>HBA2DS02</td><td>CTCCTCCCCTCCTTGCACCGGCCCTTCCTGGTCTTTGAATA</td></tr>
<tr><td>RBM8A_SNP2|C</td><td>GAGACGGCTGGTGGGAAACCGGGAAGGTGCGAGAGAAGGC</td></tr>
<tr><td>PREPL-2</td><td>GCACAGAAACATCACTGGTGGGCACTATTGAAAAAGGGCAGCCG</td></tr>
<tr><td>HBBE03A</td><td>CCTGCACTGGTGGGGTGAATTCTTTGCCAAAGTGATGGGCCA</td></tr>
<tr><td>R20PBR1REF05</td><td>CCTTCCTGCTCATCACTCTTTCCTATGTCTCCATCATGGCCGCCATCT</td></tr>
<tr><td>R20FBR1REF06</td><td>AGATGCAAGACATTTGAGAAGGGGATACATCAGGTCACCTGACTCTGTGCC</td></tr>
<tr><td>R20VBR1REF07</td><td>TGTAAGTCTCTCCTCCCACCTGGGTGGCACTGTCTGATGTGGTTCTGAAA</td></tr>
<tr><td>R20NBR1REF08</td><td>GCTCTGTGCTCATGTATCACTCTTCACTCAAAGATCACAAATCAGCTTGGCAGG</td></tr>
<tr><td>HBZU</td><td>GGACGGTGCAGAACTGGACTACAAATGCAGGAGTGACTTCCTGGG</td></tr>
<tr><td>HBASEA5U</td><td>CCAATAAATGGATGAGGACGGAGCGATCTGGGCTCTGTGTTCTCA</td></tr>
<tr><td>HBAE03A</td><td>CACTGACCCTCTTCTCTGCACAGCTCCTAAGCCACTGCCTGCTGG</td></tr>
<tr><td>HBBLPWB3U</td><td>CCTCTCATCTTTGAGTTGGAGCCTCTCCCATACCCATGTGGAGAG</td></tr>
<tr><td>HUWE1-2</td><td>TTTGCCCTAGCTCTGGAAGGCTCACTATTGCCCTGATAACCAAGC</td></tr>
<tr><td>Xq28-4</td><td>TTGACATCCTGAAAGGGTTGCAAAACCCATCACAGAGAACCCCA</td></tr>
<tr><td>Xq28-1</td><td>AACCACAGGGTTACTCGGCTTGGGGTGCCCCCTAAAGCAGATA</td></tr>
<tr><td>HBA2US01</td><td>ACCATCACTTTTCATGAGCAGGGATGCACCCACTGGCACTCCTGCA</td></tr>
<tr><td>PLP1-3</td><td>CCCAAAAACATCCTCTGAAGCCTCTCTAACCCAGGGATCCTCCTCACTCTT</td></tr>
<tr><td>PLP1-4</td><td>GTTGACTTTAAATTTTGGGCCCCTGGGCACAACTGTAGGGAACCAG</td></tr>
<tr><td>HBBCNB3D</td><td>GCCAGAAGCTCTGGAATTCTGGCTTATCGGAGGCAAGCTGTATCTTC</td></tr>
<tr><td>TAR-3</td><td>TAGCAAGAAGGGCTTATCAAGATTCTCCCATTCCTCATCCCTGGTCC</td></tr>
</table>

<table>
<tr><td>MECP2-3</td><td>CATGAAATTGTCTGGTGGGACAAAAATTGTGGGGCACTTGAGCCTA</td></tr>
<tr><td>MECP2-4</td><td>TAGCAAGGGAAGGAGCAGGCTTCAAGTTCTGCAAGCCTTTTGCA</td></tr>
<tr><td>PLP1-1</td><td>TTTTTGCCTTGATTTGGAAGGGGAATGTTCCAAGTGAGTCTTTGGGA</td></tr>
<tr><td>SLC3A1-3</td><td>GGAATGTGTGTTTCCATTGCCAGAGCCTTTCTCCGTCTGGTGAG</td></tr>
<tr><td>PREPL-4</td><td>TAAGGTTGAGTTCACGCCCACAGCTTGGGGTCTGGCAGAAGTAC</td></tr>
<tr><td>R20PBR1REF09</td><td>GGTGATTCTAGTATATTCTGGGCACTGGGTTTCAGGTAGCATGTCCATCCAAAGG</td></tr>
<tr><td>R20FBR1REF10</td><td>GGTATGGCCACTGAAAGCATAACCAGCACTGTATTCTACCCCTGTCACCAAGTC</td></tr>
<tr><td>R20VBR1REF11</td><td>GGGAGAGTTTACCTGGACACATAGGAGAGGCATGTGTGCTGTACTCCAGAATGG</td></tr>
<tr><td>R20NBR1REF12</td><td>CGCTTTGCTTCTGTGGTCTCGAGTAATTAAGTGCTGTGATGTTAGCTGCTGGT</td></tr>
<tr><td>SLC3A1-1</td><td>CTCCCTTTCCCCTGACTCATGACCTATGACCTGCTGCCTTTTGC</td></tr>
<tr><td>Xq28-2</td><td>CAGACAGTCTGGGGACAGGTCACCACTGCCCCTTAGCTGTCACT</td></tr>
<tr><td>Xq28-3</td><td>AGACGACTTCACGTTTCGGTCCTTCCCCACCTAGCACTCGTCTC</td></tr>
<tr><td>HBA1US02</td><td>GTACTTGTGTGATGGTTAGAGGACTGCCTACCTCCCAGAGGAGGTTGAATGC</td></tr>
<tr><td>HCS-3</td><td>TCCTTGATCAAAGCAGTTAATTGAAATTATCAGGCTTTGGAGGCAAATTACT</td></tr>
<tr><td>TAR-5</td><td>CCGAACAAAACACCAGTCAACGCTGATGGGCTGTCCCATCAAAT</td></tr>
<tr><td>PREPL-3</td><td>GTGCTTAGCCAACCACTGGACATTTGAGTGAGGGCACTGACAGC</td></tr>
<tr><td>Xq28-5</td><td>AGGGATGGGTCCTTGGAAAGTCCTCAACTCTCTGCAACTGCCCA</td></tr>
<tr><td>SLC3A1-2</td><td>CCTCTGAGCCAAGTAGGTGGGAAGGAGACCCTAACAGAACGGGG</td></tr>
<tr><td>HBBE02B</td><td>AGGGTACAGTTTAGAATGGGAAACAGACGAATGATTGCATCAGTGTGGAAGTCTC</td></tr>
<tr><td>HBBE01A</td><td>AGGGCAGAGCCATCTATTGCTTACATTTGCTTCTGACACAACTGTGTTCACTAGC</td></tr>
<tr><td>TAR-4</td><td>GATTCAGTTGGAAAACCCAGCAATTTAACAAAAAGGGGGAGGGGC</td></tr>
<tr><td>HUWE1-3</td><td>GGGAGGCCTACGTATCTTTAATCTGTTAATCAAGCTGATGAAAATAACTCACAATGA</td></tr>
<tr><td>HBBU30</td><td>CTATAGCTTTAGTGGATTAGAGAAAGTAGCAGAAATGAACACATATTCAGTCTGCCAGG</td></tr>
<tr><td>R20PBR1REF13</td><td>CAGAGGGATGCTCTGCATATACACAGTGGTTCTTTGCTCCCTGTGACTCTTCCC</td></tr>
<tr><td>R20FBR1REF14</td><td>AAAGGAAATGTGGTTGCCACACAGTCAGCCACATCTTCCCTATTCCTCTG</td></tr>
<tr><td>R20VBR1REF15</td><td>TGGCAGCTCGGATTCTGAAGGTAATTTTGAGACTCCTGAAGCTGAAACCC</td></tr>
<tr><td>R20NBR1REF16</td><td>CCTAGAACTCTCTGTCCCGTAATGGGTTCAGGTCATGGCAATATCACTTCTCCC</td></tr>
<tr><td>SMNc.689C>T|M</td><td>GGAAATGGAGGCAGCCAGCCTAATAGTAAGTGGGGTGGTGGTGGTG</td></tr>
<tr><td>SMNc.689C>T|W</td><td>GGAAATGGAGGCAGCCAGCTTGATAGTAAGTGGGGTGGTGGTGGTG</td></tr>
<tr><td>R20PBR2REF17</td><td>ACAGCCCACACCATGAGCTCATGGAAAGGGAAGGGGAAGTGG</td></tr>
<tr><td>R20FBR2REF18</td><td>GGTGAGTTGAGAATCATGCCAAGGAGGACACTCGGAACAGCCAGG</td></tr>
<tr><td>R20VBR2REF19</td><td>GTGAGGTTTGCCCTGGGAGTAGAGCAAGCGAAGGAAACAGCCTGC</td></tr>
<tr><td>R20NBR2REF20</td><td>GCCTTATCTGGGGACCCACGTTTTCCACCCTGGCTACACATCCTG</td></tr>
<tr><td>HBG2D</td><td>CACTTTTTCTTCGTTGAGCCCCTTCCTCGCTGCTGTGCTGC</td></tr>
<tr><td>HCS-5</td><td>GGCGCAGGAAAGAGCCTCTTAACTTGGGCAGCTGGTGAACA</td></tr>
<tr><td>TAR-1</td><td>GCTTTGGCTCCGGTGAGTGTGGGGAAAATGGGGTAGAGTGG</td></tr>
<tr><td>PMD-4</td><td>GCTGCAGCCGAACGAGAAGGTCAGGAAGAACGTGGTGCCC</td></tr>
<tr><td>HUWE1-1</td><td>ACTCCTGGATAGCCAACAGTAGCATGTGGCGGAGCTTCTCAAAG</td></tr>
<tr><td>HBAE01A</td><td>ggcactcttctggtccccacagactcagagagaacccaccatG</td></tr>
<tr><td>HUWE1-4</td><td>TTAAGGGTGGGCTGATGTCTCATGAGCTCATCGACAGCACTCC</td></tr>
<tr><td>PREPL-1</td><td>GTGACCAGGAGCCAAAGGACTCAGTGCATTCCAGAGTCACAGCG</td></tr>
<tr><td>Xq28-6</td><td>CAGCCACTTTTGTCACTTCCCCTCTAAGACCACGCCTCTCCACC</td></tr>
<tr><td>HBASEA5D</td><td>CACTAAGCGTATTGGAAGCGTGTCGGGGCTCAGGGAACTTTTCC</td></tr>
<tr><td>RBM8A_SNP1|I</td><td>CTAGTGTCTGAGCGGCACAGACGAGATCTCGATCGAAGGCGAGA</td></tr>
<tr><td>MECP2-2</td><td>TTACCAGCAGTTTGCCCTGACAGAtgtaatcccagcactttggga</td></tr>
<tr><td>RBM8A_SNP1|D</td><td>CCTAGTGTCTGAGCGGCACAGACAGATCTCGATCGAAGGCGAGA</td></tr>
<tr><td>R20PBR2REF21</td><td>TGAGTTATCAGGCAAACATCAGGAGGCTTTCAGAGGTCGAGCCACAGCT</td></tr>
<tr><td>R20FBR2REF22</td><td>GGTGGGAAAGCTCTCCTCTGGCTACTCTTTGGAGGGCCTTTCCTAGCC</td></tr>
<tr><td>R20VBR2REF23</td><td>GGCACAGACACCGTCAAAGCTTAAGGAGAACGGACCGTTGACACATGG</td></tr>
<tr><td>R20NBR2REF24</td><td>TTCTGCTGGCAGACACCATTTGTGAATGGACAGTTTTCCAGATGGTGG</td></tr>
<tr><td>HBAX2DS01</td><td>TCTCTCTTTCCTGTCTCCTCACACCCACCCCCAGAAGAGACCAAAAT</td></tr>
<tr><td>HBBE02A</td><td>TGGTCTATTTTCCCACCCTTAGGCTGCTGGTGGTCTACCCTTGGACC</td></tr>
<tr><td>HBBLPWB3D</td><td>TCTCTGTCTACACATGCCCAGTTTCCATTTGCCTCCTTGAGCCTCTC</td></tr>
<tr><td>HBATHAI5U</td><td>GCTGCCCTCAACCCCTGACAATCTCATCATCTCATATCTCAGGCATGG</td></tr>
<tr><td>HBBCNB3U</td><td>CTCAAAGCCTGAGATTTTGCCTTCCCATTAAATGCAGGTAGTTGTTCC</td></tr>
<tr><td>HCS-4</td><td>AAAGCCATGGCAACAACACTTGAAGCTGACTCATTATGCAAGTCCTTG</td></tr>
<tr><td>PMD-3</td><td>CATTTTATTTCCACCCTCAATCCACATTTCCAGATGTCTCTGCAGCAA</td></tr>
<tr><td>MECP2-1</td><td>CAGTGGAGCCATTAGGACAATGGAAAAGGAGGTACCCCTCCACCA</td></tr>
<tr><td>HBAX2US02</td><td>GGCAGTAGTATCTGCATCCCCTGACTCTCTCTCCACAGTTCCTGGGTAA</td></tr>
<tr><td>HBAX2DS02</td><td>AAAGAGAGCCTGTGGCAGTAGTTGTAGATGTAGCTGTGTTCCCTCAGCA</td></tr>
<tr><td>HBA2US02</td><td>GGTTGTAAACAGATAAACAAACTTGGCTCTGGGTAGGGAAAGGACAGGG</td></tr>
<tr><td>HCS-2</td><td>GTTAACAACAAAGTCAGTCAGAACCCAGGCTTTGAGGAGGACCAAGAAG</td></tr>
<tr><td>SLC3A1-4</td><td>TGTCCTGTTTGGTCTTGGGTGACCACAGCACAAGGACCCTTTTC</td></tr>
<tr><td>HBAX2US01</td><td>CCCTGAGTTATGGCTCAGCCCAGATCAAGAAACAATGCAAGTAGGTGGCC</td></tr>
<tr><td>HBBE01B</td><td>TCTCTGTCTCCACATGCCCAGTTTCTATTGGTCTCCTTAAACCTGTCTTGT</td></tr>
<tr><td>PMD-1</td><td>GCAATGAGGAGATGATACAGGCAGCAGATGAGCTAGAAGAGATGAAAAGAG</td></tr>
<tr><td>R20PBR2REF25</td><td>CAGCTGTTTCCTACCTACTCTGATTCCCTCAACCCCTAAACCCCTTGCC</td></tr>
<tr><td>R20FBR2REF26</td><td>GCATTCAAACAGCTTTCCGACATCACCAGCCAAGGATTTTTTTCCCCG</td></tr>
<tr><td>R20VBR2REF27</td><td>CCAAATTAGATGGAATCGCAGTGCAGCTTGCCAGGCAAACAAAGAGATT</td></tr>
<tr><td>R20NBR2REF28</td><td>TGGCTACAGTTTAGGGAGAGGGCTTTCTGAAGCCAAAATTTGCCCATTC</td></tr>
<tr><td>HBG1D</td><td>GACAGGACTTCTCAGAAACAGATGTTTTGGAAGAGATGGGAAAAGGTTCAGTG</td></tr>
<tr><td>HBA1US01</td><td>gccagacccccaggaaatgacttatcagtgatttctcaggctgttttctcctca</td></tr>
<tr><td>PMD-2</td><td>CAGGTCCTGGGATAACAGTGACTTTAGAAGACTCAAAAAAGGACATGACGAATGG</td></tr>
<tr><td>TAR-2</td><td>GGCCTCCTTCTGACATAATTCTAATCCTACAGTCTCAGAGATGCTGTTGTATCCTG</td></tr>
<tr><td>PLP1-2</td><td>GGGCCAGCTCTGAAAAGACACTCTTCCCAAGCACGTTTGCAAAG</td></tr>
<tr><td>HBBU10</td><td>AATGATTAAAGATAACAAGAGGTCTGAAAACAAAGTGACCAATTAAAATAAAAAGAAAGAGCCAAG</td></tr>
<tr><td>HCS-1</td><td>GATGCTAGAATTTGACCTGGAGCTTATATAATTAGAAGAGACACTAGTTTTCCTCATTTAAAAGCC</td></tr>
<tr><td>HBBU20</td><td>TGTATTTGTTTTGTTGGCTTTAATATGTTCCCTTTTCTTCAGAAAATGTGAGACAGCTTAAAATAAA</td></tr>
<tr><td>R20PBR2REF29</td><td>TGTGAGGTTGGCAACACAACACTTCTTTTTGTGATTAACCTCCCCAGGG</td></tr>
<tr><td>R20FBR2REF30</td><td>TCGTAGGAATGCCCCAGCTTCTGTTATTCCTGAAAGATGGAGAAGGGGC</td></tr>
<tr><td>R20VBR2REF31</td><td>CTCTAGCATCTGCCTTTCTGCTGAGAAGTCTTTTCTGTGCTTTGCCCCA</td></tr>
<tr><td>R20NBR2REF32</td><td>GAAAGTCAGAGCTGCGCTTTTTCCTGGTTTTATCCAGTCTGAGTCTCCCA</td></tr>
<tr><td>F8E14-6A_F</td><td>[FAM]CACCTTTGATTCATGACAGAATGCTT</td></tr>
<tr><td>F8E14-6A_R</td><td>GTTTCTTTTTTGTGCATCTGGTGGAATGG</td></tr>
<tr><td>F8E14-7A_F</td><td>[FAM]GGGCATATGCTCCAGTACTTCAAGA</td></tr>
<tr><td>F8E14-7A_R</td><td>GTTTCTTCCAAGCCTTCCAAGTTTTCTTCC</td></tr>
<tr><td>F8E14-8A_F</td><td>[FAM]TCTTCTCATCTTCCAGCAGCATC</td></tr>
<tr><td>F8E14-8A_R</td><td>GTTTCTTTGTTTTGGGCAAGTCTGGTT</td></tr>
<tr><td>F8E14-9A_F</td><td>[FAM]GGACTCAAAGAGATGGTTTTTCCA</td></tr>
<tr><td>F8E14-9A_R</td><td>GTTTCTTCCAGTCACTGTATGTATCTGAGGCAAA</td></tr>
</table>

Supplementary Table 3 The gene list detected by next generation sequencing

Diseases	Genes
Genetic deafness	GJB2 (NM_004004.5)
	SLC26A4 (NM_000441.1)
	12S rRNA (NC_012920)
α-Thalassemia	HBA1 (NM_000558.3)
	HBA2 (NM_000517.4)
β-Thalassemia	HBB (NM_000518.4)
Phenylketonuria	PAH (NM_000277.1)
Hepatolenticular degeneration	ATP7B (NM_000053.3)
Methylmalonic acidemia	MUT (NM_000255.3)
Methylmalonic acidemia with homocystinuria Cb1C	MMACHC (NM_015506.2)

NM accession number is a unique gene identifier that links to the GenBank record.

Supplementary Table 4 The number of each variant identified in 1915 couples			
Variants	Number of alleles		
GJB2, c.235delC, het	87		
SMN1, del, het	85		
CYP21A2, c.955C>T, het	51		
SLC26A4, c.919-2A>G, het	47		
HBA, αα/-α3.7	40		
GJB2, c.299_300delAT, het	25		
ATP7B, c.3316G>A, het	25		
ATP7B, c.2333G>T, het	23		
MMACHC, c.609G>A, het	19		
CYP21A2, c.293-13A>G, het	16		
PAH, c.728G>A, het	15		
HBA, αααanti4.2/-α3.7	14		
HBA, --SEA/αα	14		
GJB2, g.20398370-20523823del, het	13		
CYP21A2, c.844G>T, het	13		
CYP21A2, c.518T>A, het	11		
SLC26A4, c.2168A>G, het	9		
SLC26A4:c.1226G>A	9		
CYP21A2, large rearrangements, het	8		
ATP7B, c.588C>A, het	8		
ATP7B, c.2975C>T, het	8		
PAH, c.688G>A, het	7		
HBA, αα/-α4.2	7		
ATP7B, c.2621C>T, het	6		
GJB2:c.176_191del	6		
MMACHC, c.658_660delAAG, het	6		
MMACHC, c.567dupT, het	6		
PTS, c.272A>G, het	6		
PAH, c.721C>T, het	6		
STS, del, het	5		
PTS, c.84-291A>G, het	5		
PAH, c.782G>A, het	4		
ATP7B, c.2755C>G, het	4		
SLC26A4, c.916dupG, het	4		
CYP21A2, del, het	4		
MMACHC, c.482G>A, het	4		
PAH, c.1256A>G, het	4		
12sRNA, m.1555A>G, hom	4		
CYP21A2, c.293-13C>G, het	4		
ATP7B, c.3517G>A, het	4		
SLC26A4, c.1975G>C, het	3		
MUT, c.729_730insTT, het	3		

PAH, c.1174T>A, het	3		
PTS, c.259C>T, het	3		
MUT, c.323G>A, het	3		
PAH, c.1197A>T, het	3		
MUT, c.1106G>A, het	3		
SLC26A4, c.-2071_304+3801del, het	3		
ATP7B, c.2790_2792delCAT, het	3		
PAH, c.740G>T, het	3		
SLC26A4, c.1174A>T, het	3		
PAH, c.464G>A, het	3		
GJB2, c.257C>G, het	3		
PAH, c.611A>G, het	3		
PAH, c.1045T>G, het	2		
MUT, c.755dupA, het	2		
CYP21A2, c.92C>T, het	2		
PAH, c.1-4163_1-406del3758, het	2		
MMACHC, c.80A>G, het	2		
PTS, c.166G>A, het	2		
12S rRNA, m.1494C>T, hom	2		
SLC26A4, c.2027T>A, het	2		
ATP7B, c.2804C>T, het	2		
HBB, c.316-197C>T, het	2		
SLC26A4, c.1229C>T, het	2		
SLC26A4, c.1520delT, het	2		
FMR1, CGG repeat ⩾55	2		
HBB, c.52A>T, het	2		
HBA2, c.427T>C, het	2		
HBA2, c.369C>G, het	2		
PAH, c.442-1G>A, het	2		
GJB2, c.508_511dupAACG, het	2		
GJB2, c.416G>A, het	2		
PTS, c.155A>G, het	2		
ATP7B, c.1708-1G>C, het	2		
ATP7B, c.1168A>G, het	2		
ATP7B, c.3809A>G, het	2		
SLC26A4, c.754T>C, het	2		
HBA, -α3.7/-α3.7	2		
HBB, c.79G>A, het	2		
PTS, c.286G>A, het	2		
ATP7B, c.3443T>C, het	2		
Xq28, dup, het	2		
SLC26A4, c.1692dupA, het	1		
PAH:c.440C>T, het	1		
ATP7B, c.3982G>A, het	1		

PAH, c.1315+4A>G, het	1		
PAH, c.940C>A, het	1		
GJB2, c.139G>T, het	1		
GJB2, c.598G>A, het	1		
HBB, c.217dupA, het	1		
HBA2, c.40G>T, het	1		
PAH, c.1223G>A, het	1		
ATP7B, c.3884C>T, het	1		
HBB, c.126_129delCTTT, het	1		
MMACHC, c.315C>G, het	1		
ATP7B, c.3532A>G, het	1		
PAH, c.498C>G, het	1		
HBA, -α3.7/αα3.7	1		
SLC26A4, c.1595G>T, het	1		
GJB2, c.35dupG, het	1		
PAH, c.1315+6T>A, het	1		
DMD, del, het	1		
CYP21A2, c.292+1G>A, het	1		
PAH, c.1238G>C, het	1		
DMD, dup, het	1		
MMACHC, c.217C>T, het	1		
CYP21A2, c.332_339delGAGACTAC, het	1		
ATP7B, c.1543+1G>T, het	1		
CYP21A2, c.1069C>T, het	1		
MMACHC, c.394C>T, het	1		
ATP7B, c.3955C>T, het	1		
PAH, c.838G>A, het	1		
ATP7B, c.4003G>C, het	1		
MMACHC, c.271dupA, het	1		
F8, del, het	1		
SLC26A4, c.1173C>A, het	1		
HBB, c.-78A>C, het	1		
SLC26A4, c.1343C>A, het	1		
HBA2, c.377T>C, het	1		
ATP7B, c.525dupA, het	1		
PAH, c.208_210delTCT, het	1		
PAH, c.441+3G>C, het	1		
ATP7B, c.3443T>CATP7B:c.3700delG, het	1		
CYP21A2, c.[710T>A;713T>A;719T>A], het	1		
ATP7B, c.1846C>T, het	1		
HBB, c.-100G>A, het	1		
ATP7B, c.2930C>T, het	1		
SLC26A4, c.946G>T, het	1		
HUWE1, dup, het	1		

PAH, c.755G>A, het	1		
PAH, c.1222C>T, het	1		
PTS, c.118_121delTTTG, het	1		
PAH, c.1301C>A, het	1		
In total	787		
het, heterogeneous; dup, duplication; del, deletion; hom, homoplasmy			
	787		
